# Profiling of Volatile Metabolites of *Escherichia coli* Using Gas Chromatography–Mass Spectrometry

**DOI:** 10.3390/ijms26178191

**Published:** 2025-08-23

**Authors:** Karolina Żuchowska, Alicja Tracewska, Dagmara Depka-Radzikowska, Tomasz Bogiel, Robert Włodarski, Barbara Bojko, Wojciech Filipiak

**Affiliations:** 1 Department of Pharmacodynamics and Molecular Pharmacology, Faculty of Pharmacy, Collegium Medicum in Bydgoszcz, Nicolaus Copernicus University in Toruń, A. Jurasza 2 Str., 85-089 Bydgoszcz, Poland; karolina.zuchowska@cm.umk.pl (K.Ż.); alicjatracewska@wp.pl (A.T.); bbojko@cm.umk.pl (B.B.); 2 Department of Microbiology, Faculty of Pharmacy, Collegium Medicum in Bydgoszcz, Nicolaus Copernicus University in Toruń, Maria Curie-Skłodowska 9 Str., 85-094 Bydgoszcz, Poland; dagmaradepka@cm.umk.pl; 3 Department of Propaedeutics of Medicine and Infection Prevention, Faculty of Pharmacy, Collegium Medicum in Bydgoszcz, Nicolaus Copernicus University in Toruń, 9 Maria Curie-Skłodowska Str., 85-094 Bydgoszcz, Poland; t.bogiel@cm.umk.pl; 4 Department of Anaesthesiology and Intensive Care, 10th Military Research Hospital and Polyclinic, Powstańców Warszawy 5 Str., 85-681 Bydgoszcz, Poland; robert.wlodarski@10wsk.mil.pl

**Keywords:** volatile biomarkers, bacterial metabolites, *Escherichia coli*, gas chromatography–mass spectrometry (GC-MS), ventilator-associated pneumonia (VAP), breath markers, headspace analysis, volatile organic compounds (VOCs), bacteria cultures

## Abstract

Current diagnostic methods for bacterial infections in critically ill patients, including ventilator-associated pneumonia (VAP), are time-consuming, while empirical antibiotic therapy contributes to rising resistance. Bacteria-derived volatile organic compounds (VOCs) are being explored as specific biomarkers for pathogen identification and treatment monitoring. This study expands knowledge of *Escherichia coli* metabolism by identifying VOCs produced by both multidrug-resistant and susceptible strains, characterizing their temporal profiles during growth, and assessing VOC profile changes after imipenem exposure. Reference strains and 21 clinical isolates (derived from BAL samples of VAP patients) were cultured under controlled conditions. Headspace VOCs were preconcentrated using multibed sorption tubes and analyzed by gas chromatography–mass spectrometry (GC-MS), with compound identities confirmed using external standards. Sampling at seven time points over 24 h cultures revealed three VOC emission patterns: continuous release, temporary maximum, and compound uptake. In total, 57 VOCs were identified from the susceptible strain and 41 from the resistant one, with dimethyl disulfide, 2-butenal, ethyl acetate, and furan elevated in the resistant strain. Imipenem addition altered VOC production in the susceptible strain, with levels of six compounds elevated and seven reduced, while resistant profiles remained stable. Clinical isolates produced 71 VOCs, showing greater metabolic diversity and highlighting the relevance of isolate-derived VOCs in future studies.

## 1. Introduction

The persistent global use of broad-spectrum antimicrobials is a well-established risk factor for the spread of antimicrobial-resistant bacteria. According to the 2023 Annual Report on Antimicrobial Resistance Surveillance by the European Centre for Disease Prevention and Control (ECDC), resistance in *Escherichia coli* to at least one major class of antibiotics under surveillance—namely, aminopenicillins, fluoroquinolones, third-generation cephalosporins, aminoglycosides, and carbapenems—has increased by an alarming 23.7% since 2019 [1]. In this regard, in the case of a severe microbial infection, the main goal should be the immediate identification of the pathogen and the implementation of appropriate therapy based on antimicrobial susceptibility testing (AST). Unfortunately, microbiological testing is time-consuming (typically requiring a minimum of two days), and culturing microbes often involves invasive sample collection procedures. For example, diagnosing ventilator-associated pneumonia (VAP) may require bronchoalveolar lavage (BAL) collection, a notably invasive method. These limitations underscore the urgent need for non-invasive, rapid diagnostic techniques that can identify pathogens and potentially predict their antimicrobial susceptibility.

Since it has been confirmed that microorganisms, including pathogenic bacteria, produce a wide range of volatile organic compounds (VOCs) [2], assessing their profiles may provide promising prospects for clinicians for rapid and non-invasive detection of diverse infections. VOCs are mostly metabolic byproducts of bacterial aerobic and anaerobic fermentation, but may also serve as secondary metabolites for protection against antagonists and competitors or as signaling molecules for intercellular (including interspecies) communication [3]. Some VOCs are pathogen-specific, making them valuable biomarkers for identifying particular bacterial species. However, individual VOCs are often not unique to a single bacterial species, which limits their effectiveness in differentiating between specific strains or pathogens [3]. An example may be indole, which is widely known as a metabolite of *E. coli* [3,4,5,6,7,8,9,10,11,12,13,14,15,16,17] and which can also appear in cultures of *Pseudomonas aeruginosa*, especially wild-type strains [18,19]. Therefore, monitoring the complete patterns of secreted compounds, rather than a single metabolite, is essential to elucidate the origin of the causative pathogen. Moreover, tracking dynamic changes in VOC profiles could facilitate early infection detection, enabling intensive care unit (ICU) clinicians to implement timely, targeted antimicrobial treatments and monitor therapeutic efficacy—critical elements of precision medicine.

*E. coli* is one of the most extensively studied bacterial species, including the context of its volatilome, as thoroughly reviewed by Żuchowska and Filipiak [20]. Nonetheless, comprehensive studies are still needed to explore broader contexts, such as identifying commonalities and distinctions between resistant and susceptible strains, evaluating the impact of antibiotic exposure on VOC production, and the behavior of clinical isolates.

This study aimed to characterize the kinetics of VOC release or uptake in carbapenem-susceptible and carbapenem-resistant *E. coli* strains. The impact of imipenem addition on volatile organic compound (VOC) production was also investigated. Additionally, we examined whether VOC profiles emitted by reference strains were similarly produced by clinical isolates collected from patients with ventilator-associated pneumonia (VAP). Headspace gas from bacterial cultures was sampled at multiple time points, preconcentrated using sorption tubes, and immediately analyzed via gas chromatography–mass spectrometry (GC-MS). All culturing and sampling procedures were conducted under rigorously controlled ventilation conditions to follow the dynamic changes in temporal VOC concentration profiles.

## 2. Results

### 2.1. Bacteria Proliferation

**Figure 1:**
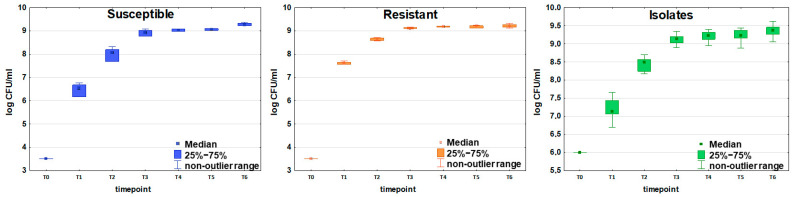
Growth curves of susceptible (blue boxes, n = 6), resistant (orange boxes, n = 5), and clinically isolated (green boxes, n = 21) strains of *E. coli*. Colony-forming units (CFU/mL) are plotted after logarithmic transformation as a function of incubation time. Timepoints T0-T6 correspond to the following periods from the beginning of the experiment: T0 = 0 h, T1 = 2 h, T2 = 3.5 h, T3 = 5 h, T4 = 6.5 h, T5 = 8 h, and T6 = 24 h. The non-outlier range defines the values below the upper limit of outlier observations (+1.0 × height of the box) and above the lower limit of outlier observations (−1.0 × height of the box).

Initial bacteria density in the culture (measured immediately after bacteria inoculation to the TSB medium) ranged from 9.15 × 10^4^ CFU/mL to 1.35 × 10^5^ CFU/mL for the susceptible strain (n = 6) and from 4.7 × 10^5^ CFU/mL to 7.2 × 10^5^ CFU/mL for the resistant strain (n = 5) as presented in Table 1. The values observed at the end of the experiment (sampling after 24 h culture) were similar for both strains and amounted to 1.61 × 10^9^ CFU/mL for the susceptible and 1.67 × 10^9^ for the resistant strain, respectively. The 24 h sampling was performed to confirm that steady bacterial growth was reached, after which the experiment was discontinued (Figure 1).

The same time points were applied in experiments on 21 clinical *E. coli* isolates. The initial bacteria density ranged from 2.60 × 10^4^ CFU/mL to 1.44 × 10^7^ CFU/mL. The mean value observed at the end of the experiment was slightly higher than for the reference strains and amounted to 2.44 × 10^9^ CFU/mL.

### 2.2. Time-Dependent Profiles of Metabolite Production by E. coli

Altogether, 57 VOCs were observed at a significantly high level in the headspace of susceptible *E. coli* compared to the pure control medium for at least one timepoint during the experiment. Among them, 44 VOCs were released and 13 were taken up by the bacteria. Similarly, 41 VOCs showed significantly different levels in the resistant *E. coli* cultures in relation to the TSB medium, out of which 34 VOCs were released and 7 were taken up by bacteria.

The headspace gas sampling timepoints were carefully selected to correspond to the bacterial growth phases and capture the dynamic changes in the emission of volatile metabolites in relation to bacterial proliferation. Based on this approach, the study identified three distinct time-dependent patterns of VOC secretion.

#### 2.2.1. Release Proportional to the Bacteria Load

In total, 29 out of 57 significant VOCs observed for the susceptible strain, and 20 out of 41 significant VOCs for the resistant strain, were released in quantities directly proportional to the bacteria load. In this model, the higher the absolute content of bacterial cells, the stronger the emission of volatile metabolites. For some metabolites (mostly alcohols), a characteristic stabilization profile was observed for the latest timepoints, fitting well with the microbes’ growth curve. Noticeably, their significantly higher abundance is already observed at the early timepoints, e.g., T3 (3-methyl-1-butanol) and T4 (ethanol, 1-propanol, 2-methyl-1-propanol), whereby the observed final abundance of alcohols is much higher than other compounds (see Table 2).

**Figure 2:**
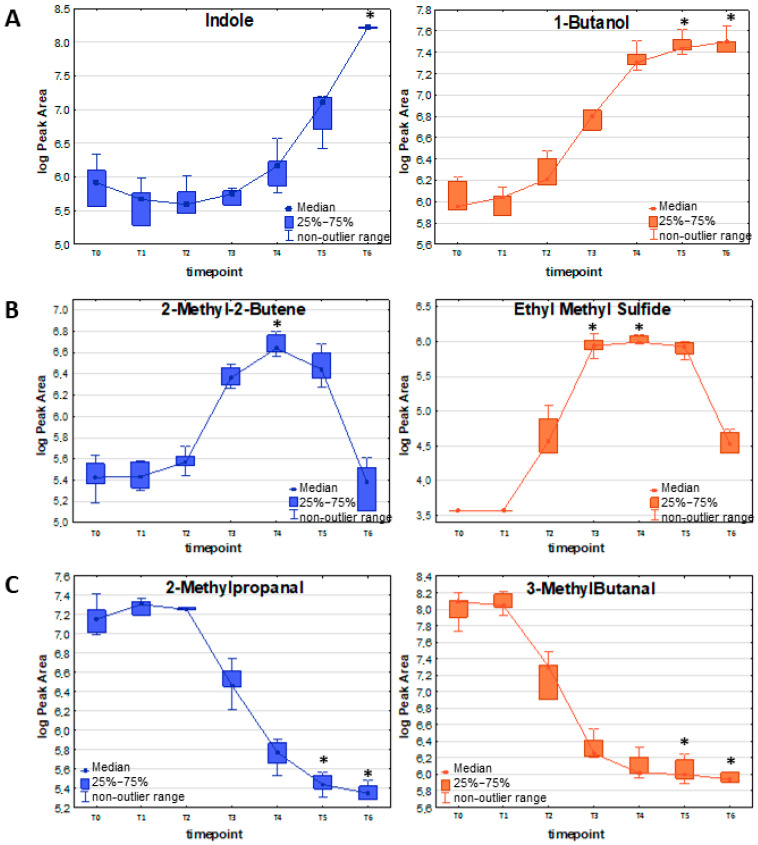
Exemplary VOC representing different release profiles in relation to the bacteria proliferation: (**A**) release proportional to the bacteria load; (**B**) release with a temporary maximum; (**C**) uptake of volatile metabolites by bacterial cells in susceptible (blue) and resistant (orange) strains of *E. coli*. The asterisk above a box indicates a statistically significant difference (Kruskal–Wallis non-parametric test to compare samples from several groups of independent observations) between VOC abundance in the respective timepoint compared to the reference medium (T0). The non-outlier range defines the values below the upper limit of outlier observations (+1.0 × height of the box) and above the lower limit of outlier observations (−1.0 × height of the box).

Other important representatives of this kinetics require substantially higher bacteria load in the culture to be finally detected at sufficiently high levels in the culture headspace. This can be well exemplified by indole, which was first detected at T4, but a significant difference in its concentration between the bacterial culture and the reference medium could only be observed at T6 (see Figure 2A and Table 2). Other metabolites released by both strains proportionally to the bacteria’s growth are some sulfuric compounds, namely methanethiol and dimethyl disulfide (DMDS), with the first significance at T3 and T5, respectively, and mercaptoacetone with first significance at T4, but with noticeably lower abundance.

Esters and hydrocarbons constitute the most numerous groups of this release pattern, with significance at T4/T5/T6. Amongst them, ethyl propionate, ethyl butyrate, and *n*-propyl propionate were not present in the TSB medium at T0; thus, they represent compounds entirely derived from *E. coli* (see Table 2).

Ketones—2-pentanone and 2-heptanone—required a 24 h bacteria proliferation to be found at adequate levels in the headspace. In turn, all three aldehydes (acetaldehyde, octanal, and decanal) were released in significant amounts much earlier, at T3 or T4 for both strains.

#### 2.2.2. Release with a Temporary Maximum

Among the 57 VOCs relevant for the susceptible strain, 15 reached the temporary maximum with the highest abundance at timepoints T3 (5 h) and T4 (6.5 h), after which a decline was observed (for the list of compounds, see Table 3). The same release profile concerned 14 out of 41 VOCs significant for the resistant ECO strain, whereby they release until reaching a maximum at timepoint T3/T4/T5, slow down, and equalize at the final point T6 roughly to the initial level in the TSB before media inoculation (T0).

The most prominent examples of this profile are volatile sulfur-containing compounds (VSCs): dimethyl sulfide, ethyl methyl sulfide (Figure 2B), dimethyl sulfone, and carbon disulfide, with their maximum abundance at T3/T4 and subsequent decrease. Also, the low-molecular hydrocarbons represent the same kinetics as 1,3-butadiene, (E-) 1,3-pentadiene (maximum at T3), isoprene (maximum at T3/T4), 2-methyl-2-butene (Figure 2B), and 2-pentene (maximum at T4/T5). Similar observations concern aldehydes—propanal, butanal, and 2-butenal—with a temporary maximum at the T3/T4/T5 timepoint.

Amongst ketones, acetone was the only compound significant for the resistant strain exclusively in T3, while (E)-3-pentene-2-one reached the temporary maximum at T1/T2, representing the earliest significant increase compared to other compounds. The only ester—ethyl n-octanoate—was not present in TSB at T0 and required a higher bacteria load to reach the maximum at T4/T5.

The timepoints corresponding to the temporary maximum vary depending on the type of VOCs and the strain; e.g., furan with the maximum at T4/T5 for the susceptible strain and T3/T4 for the resistant strain. Two compounds were significant only for the susceptible strain (1,3-pentadiene and dimethyl sulfone) and one for the resistant strain, namely carbon disulfide.

#### 2.2.3. Uptake of Metabolites

Altogether, 13 VOCs were taken up by the susceptible strain and seven compounds by the resistant strain of *E. coli* (for details, see Table 4). Apart from methyl methacrylate and five C-4 hydrocarbons (iso-butane, 2-methyl-1-propene, (Z)-2-butene, (E)-2-butene, and n-butane), the remaining VOCs, which were inversely proportional to the bacterial load, were aldehydes (Figure 2C). Among them, 2-ethylacrolein and furfural are the most efficiently used by growing bacteria of both resistance profiles, whereby a significant decline in their amount in the culture could already be observed at T4 for susceptible and resistant E.coli. Methacrolein, 3-methylbutanal (see Figure 2C), and 2-methyl-2-butenal were used more efficiently by the susceptible strain (significant decrease at T4 for the susceptible strain, and at T5 for the resistant strain). In contrast, benzaldehyde was used more efficiently by the resistant counterpart (significant decrease at T4 for the resistant strain, and at T5 for the susceptible strain).

### 2.3. VOCs Related to Antimicrobial Resistance

**Figure 3:**
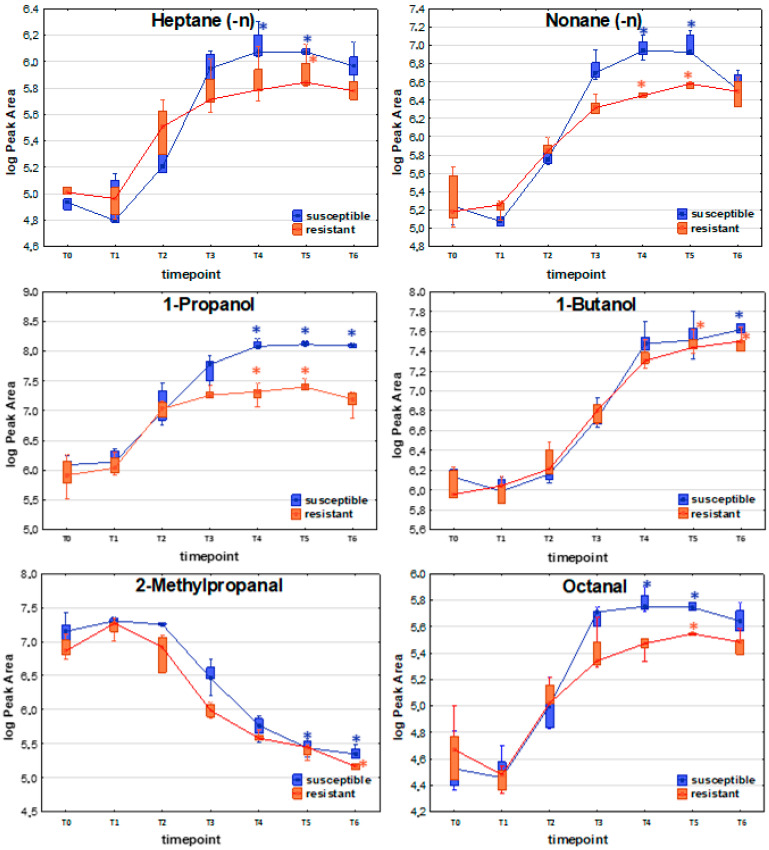
Comparison of exemplary VOC profiles released by susceptible and resistant strains of *E. coli*. The asterisk above a box indicates that a statistically significant difference (Kruskal–Wallis non-parametric test to compare samples from several groups of independent observations) was observed between VOC abundance at the respective timepoint compared to the reference medium (T0).

To determine whether resistant and susceptible *E. coli* strains produce significantly different profiles of volatile metabolites, the Mann–Whitney U test was employed to compare VOC levels at corresponding timepoints during bacterial growth. Based on the three patterns outlined in the previous section, the analysis was restricted to timepoints reflecting discrepancies between the maximum metabolic activity of both strains, namely, T3 or T4 (temporary maximum profile) and T5 and T6 (compounds with profiles directly proportional to bacterial load and compounds taken up during bacteria proliferation). Only metabolites that exhibited significantly different levels in the culture of at least one strain (either resistant or susceptible) compared to the reference medium were included in the comparison. A comparison of profiles of exemplary VOCs released by susceptible and resistant strains of *E. coli* is presented in Figure 3.

Among compounds with the release profile directly proportional to the bacteria load, significantly higher levels in susceptible (compared to resistant) strains were observed for 1-propanol (T3, T4, T5, T6), acetaldehyde (T6), ethyl propionate (T4, T5, T6), n-propyl propionate (T5, T6), and indole (T4, T5, T6).

For the temporary maximum release profile, VOCs that were found at significantly higher levels for the susceptible strains comprised 1,3-butadiene (T4), (E)-1,3-pentadiene (T4), propanal (T3, T4, T5), 2-butenal (T4), ethyl n-octanoate (T4), and furan (T2).

Compounds taken up by bacteria with significantly higher levels in susceptible *E. coli* are represented by C-4 hydrocarbons (isobutane, 2-methyl-1-propene, (Z)-2-butene), with a significant difference at T2. Another representative group comprises four aldehydes—methacrolein (T3), 2-methylpropanal (T2, T3), 3-methylbutanal (T2), and benzaldehyde (T3, T4)—all of which were found at significantly higher levels for the susceptible *E. coli* strain.

The distinctive compounds were ethyl acetate (T5), dimethyl disulfide (T5), 2-butenal (T2), and furan (T2). These four compounds were the only VOCs that showed statistically significant differences between the susceptible and resistant *E. coli* strains at a given timepoint, with significantly higher levels observed in the resistant strains.

Noticeably, the abundance of released VOCs was slightly higher for the susceptible strains than the resistant strains. Due to this reason, several compounds were found to be statistically significant solely for susceptible *E. coli*, for instance, pentane, 1-hexene, undecane, 2-pentanone, 2-heptanone, n-propyl propionate, n-butyl acetate, 3-methylbutyl acetate, mercaptoacetone, and indole. These compounds were released by both strain types, but a statistically significant difference was reached only for the susceptible strain due to the sufficiently high levels detected in the headspace. In the resistant strain, their concentrations were too low to reach differences of statistical significance.

### 2.4. Effect of Antibiotics on VOC Release by E. coli

To examine the impact of carbapenem on VOC secretion from bacteria, 0.5 mL of an imipenem solution (2.5 mg/L in PBS) was added to the bacterial culture at timepoint T2 (i.e., 3.5 h post-inoculation), following headspace gas preconcentration onto a sorption tube.

In principle, antibiotics are expected to exert a substantially stronger effect on susceptible rather than on resistant strains, which may also influence the production of volatile metabolites. Based on the observations of the growth profiles of the susceptible and resistant strains with and without the addition of imipenem, it was found that the addition of the antibiotic at point T2 caused a bactericidal effect for the susceptible strain and did not significantly affect the proliferation of the resistant strain producing beta-lactamases (see Figure S1 for details).

Considering VOCs released by each strain separately (as presented in Table 2, Table 3 and Table 4), no metabolites were released at significantly higher levels in the resistant *E. coli* culture after the addition of imipenem. In contrast, 29 compounds were released in significantly lower amounts following the addition of imipenem to the culture. Eighteen compounds remained unchanged, while 12 other compounds were released in significantly lower quantities compared to the same timepoint without imipenem, although they were not significantly altered in the pure resistant strain (Table 5).

**Figure 4:**
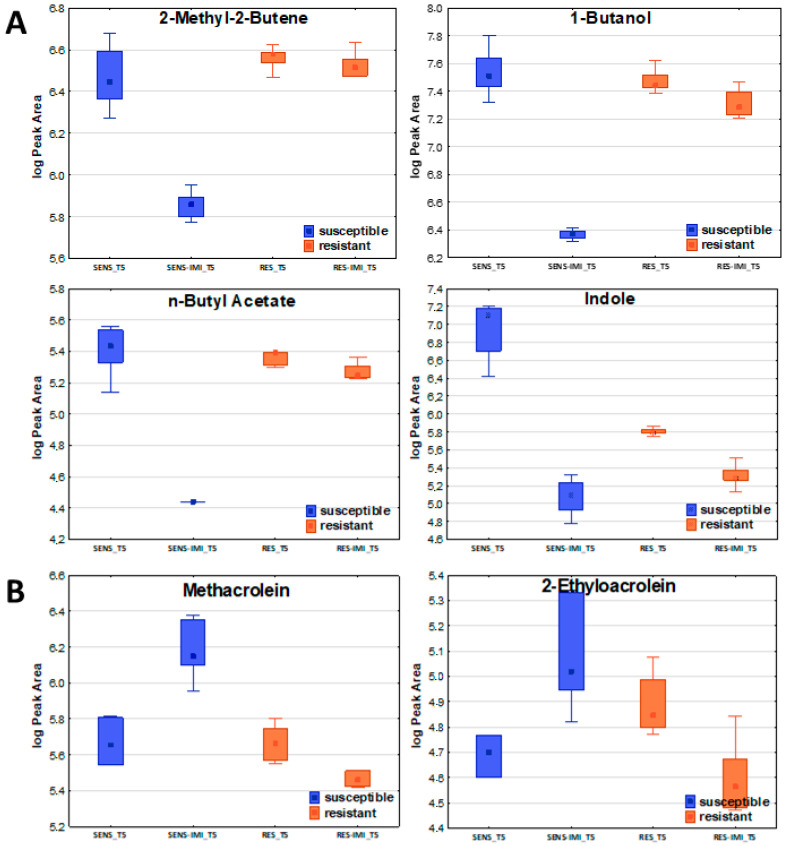
The effect of imipenem on metabolite secretion from *E. coli*. A significant decrease (**A**) or increase (**B**) in VOC amounts in bacterial headspace was found for susceptible strains (blue boxes on the left) but not for resistant ones (orange boxes on the right).

Concerning the susceptible strain, six compounds revealed a significant increase after the addition of imipenem: E-2-butene, methacrolein, 2-methylpropanal, 2-ethylacrolein, 3-methylbutanal, and benzaldehyde. The abundance in bacterial headspace of 31 out of 57 VOCs significant for the susceptible strain substantially decreased after the addition of imipenem (Figure 4). In total, 21 compounds were unchanged under these conditions, as there were no significant differences in the non-parametric Mann–Whitney U test for their level at timepoint T5 without and after imipenem implementation. Among the 31 volatile organic compounds (VOCs) that were significantly reduced in the susceptible strain, 7 remained unchanged in the resistant strain, making them particularly interesting as potential markers or contributors to antibiotic resistance. The complete list of compounds affected and unaffected by the addition of antibiotics to the bacteria culture is given in Table 5.

### 2.5. VOCs Released from Clinical Strains of E. coli

VOC secretion profiles for 21 clinical strains isolated from BAL specimens collected from VAP patients were determined to compare the metabolites released by reference and wild *E. coli* strains. Since none of the isolates were imipenem-resistant, all clinical strains were referred to the antimicrobial susceptible reference strain.

**Figure 5:**
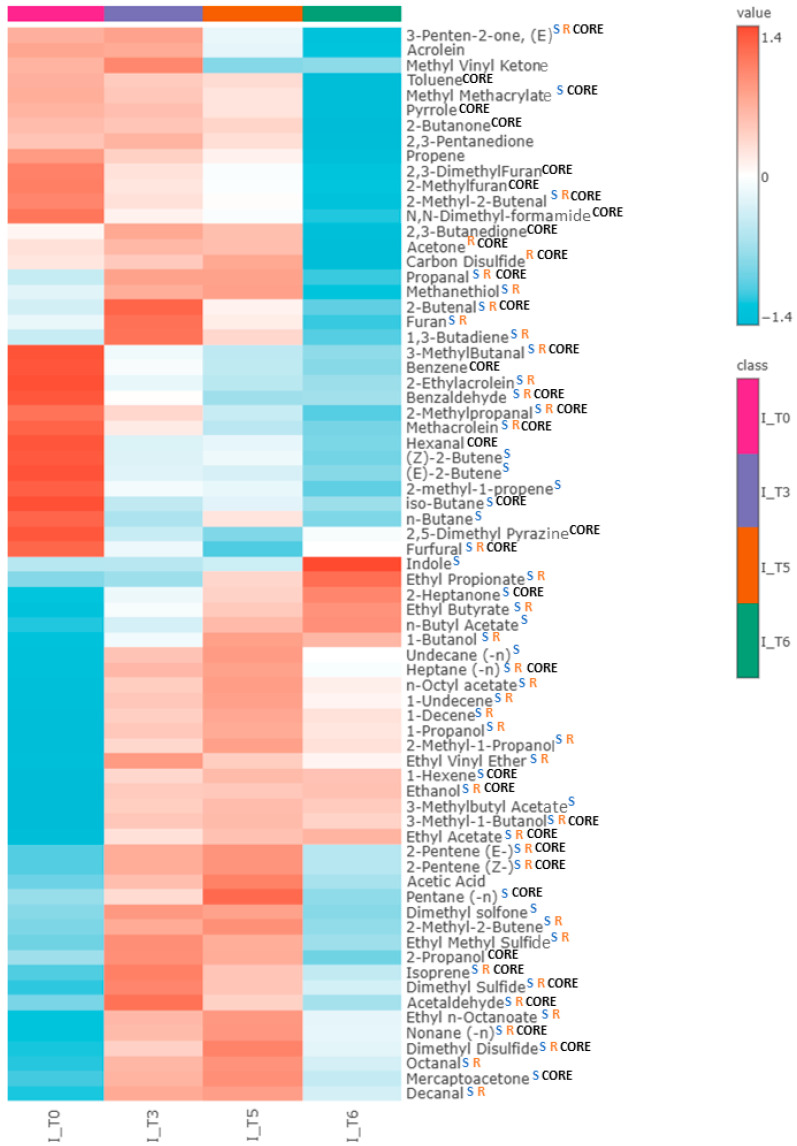
Heatmap representing the average abundance of all metabolites significantly released by clinical isolates of *E. coli* (for T0, T3, T5, n = 21, while for T6, n = 16) at the respective timepoint of bacterial growth: T0 (0 h), T3 (5 h), T5 (8 h), T6 (24 h). Blue “S” indicates compounds significant for susceptible strain, orange “R” indicates compounds significant for resistant strain, and black “CORE” indicates core volatilome—compounds present in all clinical isolates at every timepoint. Compounds not labeled with “CORE” consist of pan-volatilome.

Altogether, 71 VOCs were found to have a significantly different level compared to pure TSB medium. The aforementioned three profiles of VOC production were observed (Supplementary Figure S2). In total, 28 out of these compounds were released proportionally to the bacteria load, 16 showed a temporary maximum, and another 27 were taken up by proliferating cells (Table 6). Notably, 39 of the released VOCs were detected at all timepoints across all clinical isolates tested, suggesting that they can be considered a “core volatilome” of wild-type *E. coli* strains. The compounds without full occurrence can be defined as “pan volatilome”. In the case of wild *E. coli* strains, “pan volatilome” consists mainly of hydrocarbons and esters, e.g., ethyl propionate (n = 0/21 at T0, n = 3/21 at T3, n = 16/21 at T5 and n = 15/16 at T6), ethyl n-octanoate (n = 0/21 at T0, n = 20/21 at T3, n = 20/21 at T5 and n = 12/16 at T6), and n-octyl acetate (n = 1/21 at T0, n = 19/21 at T3, n = 20/21 at T5 and n = 13/16 at T6). Other compounds that do not occur in all *E. coli* isolates are indole (n = 20/21 at T0 and T3), 1-propanol (n = 20/21 at T0), 2-methyl-1-propanol (n = 15/16 at T6), 1-butanol (n = 11/21 at T0, n = 20/21 at T3), and sulfuric compounds—methanethiol (n = 15/16 at T6), ethyl methyl sulfide (n = 0/21 at T0, n = 20/21 at T3, n = 20/21 at T5 and n = 6/16 at T6), and dimethyl sulfone (n = 12/21 at T0, n = 8/16 at T6). The overview of the VOC abundance in clinical isolates at the chosen timepoints of bacteria growth is given in the form of a heatmap (Figure 5).

Notably, out of 57 VOCs significant for the susceptible strain, 45 were also significant for the clinical isolates. Twenty-five VOCs significant for susceptible *E. coli* are also part of the core metabolome of clinical isolates. Additionally, two compounds significant for the resistant reference *E. coli*, acetone and carbon disulfide, were released in clinical isolate cultures. Among the remaining twenty-six compounds, significant only for clinical isolates, five were released in relation to the bacterial load, six were released with a temporary maximum, and fifteen were taken up. The complete set of compounds metabolized by clinical isolates of *E. coli* is presented in Table 6 and Figure 5.

## 3. Discussion

In an ideal clinical setting, managing ventilator-associated pneumonia (VAP) and other severe infections would rely on the prompt initiation of targeted antibiotic therapy, guided by the identification of the causative pathogen and its antimicrobial susceptibility profile. In reality, however, initial treatment typically involves broad-spectrum empirical antibiotic administration. This approach is necessitated by the time required for conventional microbiological analyses—usually 48 to 72 h—to accurately identify the pathogen and determine its susceptibility pattern. Moreover, obtaining appropriate clinical specimens for microbiological testing often requires invasive procedures, such as bronchoscopy with bronchoalveolar lavage (BAL), particularly in the context of VAP.

As a result, over the past several years, significant research efforts have been dedicated to developing diagnostic methods that enable rapid and direct detection of specific pathogens, facilitating the immediate implementation of appropriate pathogen-targeting antibiotic therapy. One promising approach involves the analysis of volatile organic compounds (VOCs), which are metabolic byproducts emitted by all living organisms. These VOCs can serve as unique biochemical signatures, allowing for the differentiation of microbial species. VOCs can be detected not only in vitro but also in vivo (e.g., via breath analysis) [28,29,30] and ex vivo (e.g., from blood [31,32], sputum [33,34,35], BAL [36,37,38], or urine [39] samples), providing real-time insights into ongoing disease processes within the host. The specific profile of VOCs produced by a given microorganism—a “volatile fingerprint”—comprises characteristic, though not necessarily unique, compounds that reflect the organism’s metabolic state. The detection of VOCs enables the identification of bacterial species and can even distinguish between phenotypes within the same species, for instance, differentiating between antibiotic-resistant and susceptible strains [20,40].

Despite substantial evidence supporting the non-invasiveness, rapidity, and potential clinical utility of microbial VOC analysis, no biomarker has yet been validated to achieve clinical relevance for reliably predicting the onset of bacterial infections in the lungs, urinary tract, or sepsis. This is mainly due to limitations inherent in existing studies, including small sample sizes and the frequent restriction to single-center investigations. Such constraints can introduce bias and increase the risk of drawing erroneous conclusions, such as mistaking exogenous contaminants for potential biomarkers. Accurate interpretation of data requires the correct identification of the target metabolite instead of reporting unspecified “features”. The bacterial origin of the compounds of known structures can be confirmed through in vitro model studies using microorganisms. When chromatographic techniques are used, this process should involve analysis of reference standards for VOC identification, rather than relying solely on preliminary matching of unknown spectra to entries in mass spectral libraries. Precise identification of the putative biomarker allows for elucidating the biochemical pathways responsible for its production in bacterial cells, thereby enhancing the interpretability and specificity of clinical study results. The VOC libraries characteristic of specific bacterial species, obtained through in vitro analyses, should subsequently be validated in a clinical setting—for example, by analyzing exhaled breath samples from patients with confirmed VAP. In our recently published pilot study [28] based on breath analysis, we achieved a diagnostic performance of 80–90%, with results such as an AUC of 0.893, a sensitivity of 0.870, and a specificity of 0.824. These findings position breath analysis as a competitive method compared to currently used non-invasive diagnostic approaches for VAP, such as clinical scoring systems or biomarkers (e.g., procalcitonin or CRP). In our opinion, to implement breath analysis as a tool for VAP diagnosis in clinical practice, it is essential to build comprehensive VOC libraries specific to each pathogen, elucidated from in vitro experiments with pathogenic strains, rather than focusing on VOCs found in breath gas without consideration of their origin (what could erroneously include. e.g., exogenous contaminants or endogenous compounds, but not related to bacteria presence). The crucial step should always be extensive multicenter studies on large patient cohorts and comparison with standard diagnostic methods (e.g., microbiological culture from BAL) to confirm its clinical utility.

In this study, reference *E. coli* strains—both carbapenem-susceptible and carbapenem-resistant—were examined under controlled in vitro conditions to investigate the kinetics of VOC emission and uptake, as well as to identify metabolites potentially linked to antibiotic resistance. Furthermore, the impact of antibiotic exposure on VOC production was evaluated. Ultimately, the VOC profiles of these reference strains were compared to those of clinical *E. coli* isolates collected from patients with ventilator-associated pneumonia.

### 3.1. Time-Dependent Profiles of Metabolite Production by E. coli

Dynamic changes in microbial VOC production kinetics result from the total bacterial load and the metabolic activity of the cells. Importantly, these changes may have clinical relevance: metabolites released during the early phases of bacterial growth could indicate emerging infections. Some of these compounds, such as sulfur-containing volatiles (hydrogen sulfide, methyl mercaptan, dimethyl sulfide, dimethyl disulfide, and dimethyl trisulfide), are suspected—due to their high toxicity—of directly contributing to the development of inflammation [40]. Conversely, compounds whose release correlates directly with bacterial load may be helpful in monitoring the course of an infection, providing insight into disease progression or resolution, for example, in response to antibiotic treatment. Such compounds may include 2-pentanone and dimethyl sulfide (DMS), which are released during the early stages of bacterial growth. A decrease in their exhaled concentrations has been observed during the resolution of VAP in preliminary clinical studies [41].

#### 3.1.1. Release Proportional to the Bacteria Load

Among the compounds whose release patterns were associated with bacterial load, alcohols (n = 5) represented the most prominent group, with significance at timepoints T4–T6 for both reference strains, suggesting a strong correlation between their production and the proliferation of bacterial cells. Terminal alcohols (ethanol, 1-propanol, and 1-butanol) are produced through β- or α-oxidation of fatty acid derivatives. Although ethanol is one of the most studied microbial volatiles, described as a metabolite strongly related to *E. coli* growth [8,15,24], probably depending on glucose availability [21], it does not make it a potential marker, as it has also been observed in *Klebsiella pneumoniae, Staphylococcus aureus*, and *Streptococcus pneumoniae* cultures [40]. 1-Butanol, significant in our study for both the resistant and susceptible strains, has been previously reported as potentially related to resistant *E. coli* [25], released regardless of culture media type [9]. 1-Propanol [16,24] has been reported to be most prominently released by *E. coli* strains—however, not exclusively, because it has also been reported for *K. pneumoniae* [3]. Our observation is consistent with the findings of Zheng et al. [25], as they also noted that 3-methyl-1-butanol is emitted by both the sensitive and the resistant strains of *E. coli*. Both 3-methyl-1-butanol and 2-methyl-1-propanol have been discussed as volatile metabolites of *E. coli* less often than other VOCs, and they are found somewhat less frequently in *E. coli* compared to other pathogens [10,40].

Intriguing profiles were observed for aldehydes, whereby among low-molecular (highly volatile) ones, only acetaldehyde [12,16,25] was released by both strains proportionally to the bacteria load, with the concentration difference reaching statistical significance at T6. The remaining aldehydes released proportionally to bacterial growth were octanal [23] and decanal [14,23], both of which were previously mentioned as related to the *E. coli* cultures. Ketones (2-pentanone and 2-heptanone), which were significantly increased at T6 only for the susceptible strain, most probably originate from the β-oxidation of fatty acids [42]. Similarly, 2-heptanone was used as a discriminative compound by Drabińska et al. [22] for the differentiation between CTM-X extended spectrum beta-lactamase positive and negative *E. coli* strains, with an accuracy of 89.9%. However, Chen et al. observed that 2-heptanone was released with a temporary maximum point by *E. coli* O157:H7 [4]. Both compounds were detected in the headspace of *E. coli* in similar experiments [14,26,27], while in previous work, we reported their production in *K. pneumoniae* cultures [43], which is also confirmed in the literature [44,45].

The most interesting compound released according to this profile that could potentially become a biomarker of *E. coli* infection is indole, the product of the conversion of tryptophan by tryptophanase [46]. Although indole has been confirmed as a product of *E. coli* in numerous independent in vitro studies using various analytical platforms—GC-MS [3,4,5,7,8,10,14,17], MCC-IMS [6,13], GC-IMS [9], PTR-MS [11], and SIFT-MS [12,15,16,17]—other studies have shown that it is not unique for *E. coli*, as it is also released by *S. aureus* [7], *K. pneumoniae, A. baumanii* [47], and *P. aeruginosa* [33,47,48]. However, in the case of *E. coli* strains, the production of indole is much higher [49] than for other bacteria species, which can be useful in differentiating *E. coli* from other bacteria [11,13,14,15].

Among esters, ethyl acetate and ethyl butyrate remain the most promising VOC characteristics for *E. coli*, since these bacteria have been reported to have the most prominent production of acetate-containing esters [40]. Methanethiol and dimethyl disulfide are VSCs produced by almost all Gram-negative bacterial species, including *E. coli, K. pneumoniae*, and *P. aeruginosa* [6,15,40].

The remaining compounds exhibiting this secretion profile were hydrocarbons (n = 7), esters (n = 4), mercaptoacetone, and ethyl vinyl ether. None of them was reported as a volatile metabolite of *E. coli* in previous studies.

#### 3.1.2. Temporary Maximum

In the second VOC emission profile observed in bacteria, the highest levels of metabolites occurred at timepoints T3 and T4, which align with the latter part of the logarithmic growth phase of *E. coli* under the given conditions. This suggests that the production of VOCs following this pattern is primarily driven by intense cellular activity—such as bacterial cell division—rather than by overall bacterial density. Thus, the dynamics of metabolic processes, not the absolute number of bacteria, play a key role in VOC release in this profile.

This group comprises a relatively small number of compounds, including hydrocarbons (n = 6), aldehydes (n = 3), volatile sulfur compounds (VSCs, n = 4), furan, ethyl n-octanoate, and two ketones: acetone and (E)-3-penten-2-one. Among the hydrocarbons, isoprene is the only compound previously linked in the literature to the presence of *E. coli* [9,21]. It is believed to originate from the transformation of dimethylallyl pyrophosphate or 3-methyl-2-buten-1-ol [50]. However, it should be noted that isoprene is also one of the main volatile compounds in the breath of mammals [51], which is why its usefulness in in vivo studies is negligible [40]. The presence of butanal [9,22] was in agreement with the existing literature; however, Shestivska et al. [48] also observed the release of this metabolite from *P. aeruginosa*. A statistically significant amount of carbon disulfide (CS2) was observed only for the resistant *E. coli* strain in our study. This metabolite was mentioned only twice as potentially released by *E. coli* [9,21]; thus, further studies on this compound are needed. Dimethyl sulfide and ethyl methyl sulfide were also detected by Smart et al. [23] as more likely produced by susceptible *E. coli* isolates. The major mechanism for the formation of sulfuric compounds is the degradation of S-containing sulfolipids or amino acids (methionine, cysteine, and their derivatives) [52]. Acetone, which achieved a level of statistical significance only for the resistant strain, has been reported as a compound closely associated with bacterial growth and metabolism [25]. Acetone is considered an *E. coli*-related mVOC [23,25], but similarly to isoprene, it is present in high concentrations in breath, which limits its in vivo applicability as a biomarker for bacterial infection.

Metabolites showing a temporary maximum in their release profile reach high concentrations during the early stages of bacterial growth. This characteristic makes them promising candidates for further investigation as potential early markers of developing bacterial infections.

#### 3.1.3. Uptake of Metabolites

An important finding of this study is the clearly marked uptake of aldehydes beginning simultaneously with the logarithmic phase of bacterial growth. Abundances of methacrolein, 2-methylpropanal, 2-ethylacrolein, 3-methylbutanal, 2-methyl-2-butenal, furfural, and benzaldehyde decrease inversely proportional to the total amount of bacteria. Aldehydes are end products of amino acid metabolism and intermediate products for the formation of many esters [40]. This clearly indicates that the aldehydes present in the composition of the TSB medium serve as a source of energy for the multiplying bacteria. Aldehydes also act as intermediates in alcohol synthesis [53], which explains their noticeable decrease during the experiment, coinciding with the exponential increase in alcohol release.

### 3.2. VOCs Related to Antimicrobial Resistance

In the context of compounds potentially associated with antibiotic resistance, only four compounds were observed at significantly higher levels in *bla*_VIM_-positive isolate *E. coli* cultures (Verona-integron encoded beta-lactamase) compared to the susceptible strain. These compounds—2-butenal, ethyl acetate, dimethyl disulfide, and furan—are not well-characterized in the literature; however, there are some reports of dimethyl disulfide and ethyl acetate release by *E. coli* [5,6,14,15,16,22,23]. Unlike our findings, Smart et al. [23] observed that dimethyl disulfide (DMDS) was significantly associated with susceptible isolates, and its emission from resistant isolates decreased in relation to susceptible isolates following cephalexin addition. Such differences could serve as potential markers of antibiotic resistance; however, further research is needed to confirm their diagnostic value.

### 3.3. Effect of Antibiotics on VOCs Released by E. coli

It has been previously demonstrated that antibiotics can significantly affect the bacterial metabolome [16,23,25,43]. Imipenem is a beta-lactam antibiotic that inhibits cell wall synthesis [54]. In clinical practice, antibiotic dosing is often based on achieving concentrations that exceed the MIC for the target pathogen to ensure efficacy. For imipenem, the concentration we selected corresponds to an MIC above those for sensitive *E. coli* strains typically encountered in clinical settings but below the value for the resistant ones. Furthermore, we decided to test this boundary antibiotic concentration in our study, which could have provided additional insight into the dose–response effects. However, we selected the dose because it is within the therapeutic range observed in clinical use, where imipenem effectively inhibits the bacterial growth of susceptible strains without inducing excessive toxicity. In future studies, we plan to explore a wider range of antibiotic concentrations, including a wider range of doses, to better understand the dose-dependent relationships and the potential for metabolic changes at varying levels of bactericidal drug exposure.

In our study, the addition of imipenem to the bacterial culture led to a clear shift in the VOC profile, marked by a significant reduction or increase in VOC levels, presumably due to the killing of susceptible cells. Moreover, dead bacteria can release various substances from the intracellular matrix into the environment.

Six compounds normally taken up by the bacteria were significantly elevated in the headspace of susceptible *E. coli* culture after imipenem addition, while in the resistant one, they retained their original profile (Table 5). These compounds included E-(2)-butene, methacrolein, 2-methylpropanal, 2-ethylacrolein, 3-methylbutanal, and benzaldehyde. Most of these are aldehydes, which, as previously mentioned, serve as a source of nutrients in the medium, thereby supporting bacterial growth. In the case of susceptible bacteria, the addition of imipenem leads to cell death, which results in the inhibition of the initial compound uptake from the medium and the equilibration of its level in culture headspace—hence the observed increase in their emission. The emission of the other seven compounds (2-methyl-2-butene, Z-(2)-pentene, E-(2)-pentene, 1-butanol, n-propyl propionate, n-butyl acetate, ethyl n-octanoate), normally released by both reference strains, significantly declined for the susceptible strain after the addition of imipenem, with no change in the profile for the resistant one. The observed decrease in VOC emissions by the susceptible strain following imipenem treatment, alongside the unchanged levels of the same VOCs in the resistant strain, indicates that the antibiotic effectively disrupts metabolic pathways in the susceptible strain. In contrast, the stable VOC profile in the resistant strain suggests that it is able to maintain its metabolic functions despite imipenem exposure.

Previously, three studies on the impact of antibiotics on VOC emission in *E. coli* have been published [16,23,25]. A comparable investigation was conducted by Zheng et al. [25], who assessed the impact of imipenem addition on carbapenem-susceptible and carbapenem-resistant *E. coli*. Our findings share several similarities with those reported by Zheng et al. Specifically, after imipenem treatment, both acetaldehyde and benzaldehyde were detected at reduced levels in the resistant strain relative to the susceptible strain, while 1-butanol was emitted in greater amounts by the susceptible strain. Allardyce et al. [16] investigated the changes in VOC emission from the same susceptible strain of *E. coli* (ATCC 25922) after gentamycin treatment. They also observed a significant decrease in the production of VOCs (acetaldehyde, ethanol, DMDS, DMS, indole, methanethiol, and propanol) after gentamycin addition, regardless of the dosage above or below the minimal inhibitory concentration (MIC). Smart et al. [23] found that, following cephalexin treatment, several compounds investigated in our study—ethanol, acetone, DMS, octanal, and decanal—were more likely present in the cephalexin susceptible strain than in the resistant one. Taken together, the results of our study and previous investigations consistently demonstrate that antibiotic treatment leads to specific and reproducible changes in the VOC profiles of *E. coli,* with apparent differences observed between susceptible and resistant strains. These findings underline the potential of VOC analysis as a complementary approach for understanding bacterial responses to antibiotic pressure and possibly for future diagnostic applications.

### 3.4. VOCs Released from Clinical Strains of E. coli

To determine whether the metabolites produced by reference strains under in vitro conditions are also relevant to clinical strains causing VAP in ICU patients, the VOC release profiles were analyzed for 21 *E. coli* isolates obtained from BAL samples. Thirty-nine out of seventy-one VOCs were found to have 100% occurrence in clinical samples; hence, they stand as the “core-volatilome” of clinical *E. coli* strains. Moreover, most of them (27 out of 39) were also observed for the reference *E. coli* strains.

Previous studies have identified several VOCs as characteristic of *E. coli*, including indole [3,4,5,6,7,8,9,10,11,12,13,14,15,16,17], 2-nonane [4,8], 2-heptanone [4,22], acetic acid [3,14], 2-tridecanone [14,46], 2-pentadecanone [14,46], 1-octanol, and 1-hexadecanol [8,14,46]. The GC-MS configuration, particularly the choice of chromatographic column, substantially affects the range of detectable VOCs. For instance, while using the Q-bond column in our study ensures the superior chromatographic separation of relatively low-molecular analytes, it simultaneously precludes the detection of heavier compounds such as tridecanone, pentadecanone, and hexadecanol. Out of the previously reported, only indole, acetic acid, and 2-heptanone have been detected in a group of strains included in our study. Noticeably, none of the compounds observed in our research is released exclusively by *E. coli*, as they were also confirmed in the cultures of *K. pneumoniae*, *S. aureus*, and *P. aeruginosa* [6,15,40]. This may hinder the ability to distinguish between bacteria species; therefore, it seems necessary to identify and investigate combinations of several dozen VOCs that could together serve as a bacterial biochemical fingerprint.

### 3.5. Study Limitations

A relatively small number of isolates and their origin from a single center are features limiting this research. Another limitation of this study is the lack of carbapenem-resistant strains among clinical isolates. More investigations are needed to examine the impact of carbapenem on VOC levels in strains isolated from patients. In addition to the putative metabolic mutations in the wild-type clinical strains, they are frequently exposed to various antimicrobial agents in the hospital setting, particularly during active infection. In contrast, the reference strains are maintained and cultured under consistent, controlled laboratory conditions. As a result, clinical strains may display distinct metabolism and growth kinetics, potentially accounting for variations in growth curves and VOC emission or uptake profiles. The Q-bond column applied in this study (see Section 4.4) further limits the range of analyzable compounds to relatively low-molecular, highly volatile substances. Still, it was crucial to ensure the superior chromatographic separation of the vast number of coexisting metabolites, which is required for their unequivocal identification—one of the fundamental objectives of this work.

## 4. Materials and Methods

### 4.1. Bacteria Cultivation

Carbapenem-susceptible *E. coli* strain (ATCC 25922) was purchased from the American Type Culture Collection (strain 25922, ATCC, Manassas, VA, USA), and carbapenem-resistant *E. coli* strain, *bla*_VIM_-positive isolate, was obtained as a generous gift from the collection of the Department of Clinical Microbiology at the University Hospital No. 1 in Bydgoszcz, Poland. The resistant *E. coli* used in this study was confirmed to produce the VIM-type beta-lactamase enzyme. The susceptibility of both strains was confirmed using a BD Phoenix™ M50 instrument (Becton, Dickinson and Company, Sparks, MD, USA) with BD Phoenix™ NMIC-402 panels (Becton, Dickinson and Company, Sparks, MD, USA). Additionally, the tested strains were also phenotypically checked for the presence of clinically important carbapenemases (oxacillinases, metallo-beta-lactamases and *Klebsiella pneumoniae* carbapenemases), interpreted according to EUCAST Recommendations (Version 13.0).

For the VIM-like metallo-beta-lactames-positive *E. coli* strain, molecular analysis was performed, checking for the presence of specific resistance genes (NDM, VIM, KPC, IMP, OXA-181, and OXA-48 types) using a commercially available kit (eazyplex^®^ SuperBug complete, AmplexDiagnostics GmbH, Gars-Bahnhof, Germany).

Both bacterial strains were suspended in a tryptic soy broth (TSB) medium at a stirring rate of 80 rpm and grown in a 3D culture. Plating for colony-forming unit (CFU) counts was performed on Difco ^TM^ MacConkey Agar plates (Becton, Dickinson and Company, Franklin Lakes, NJ, USA).

The same protocol was used to culture 21 clinical isolates of *E. coli* obtained from BAL samples taken from mechanically ventilated patients suffering from VAP hospitalized in the Anesthesiology and Intensive Care Unit of the 10th Military Research Hospital and Polyclinic in Bydgoszcz, Poland. The samples were first quantitatively plated on various culture media following standard microbiological procedures. After overnight incubation at 37 °C, a qualitative read-out was conducted. Strains reaching a specified threshold were selected for antimicrobial susceptibility testing and screening for major antimicrobial resistance mechanisms. Subsequently, these strains were re-cultured on non-selective media and stored at −80 °C in Brain Heart Infusion broth with 10% glycerol for further analysis.

### 4.2. Headspace Sampling

A custom-built system, described in detail elsewhere [43,55], was used to sample headspace gas from bacterial cultures. Briefly, the glass bottles containing 100 mL of bacterial suspension in tryptic soy broth (TSB) were incubated at 37 °C in a water bath inside an incubator set to 45 °C (to maintain temperature stability and minimize condensation in gas transfer lines). Synthetic air with a purity of 6.0, enriched with 5% CO_2_ (Air Products, Diegem, Belgium), was further purified using a Supelcarb filter (Supelco, Bellefonte, PA, USA). The purified air was split into two lines: firstly, a low-flow line (5 mL/min), which passed the water purge (humidifier) and subsequent bacterial culture; secondly, a high-flow line (40 mL/min), used to reduce the relative humidity of the transferred bacterial headspace gas. Flow rates were precisely controlled using mass flow controllers with automatic compensation (Vögtlin Red-Y smart series, NewTech, Gliwice, Poland) and additionally verified during headspace sampling using a mass flow meter (Vögtlin Red-Y Compact, NewTech, Gliwice, Poland) installed at the system outlet. Sorption tubes were packed with 140 mg of Carbotrap B (20/40 mesh) and 330 mg of Carbotrap X (60/80 mesh), enabling efficient capture of a wide range of volatile organic compounds (VOCs) while minimizing water adsorption on hydrophobic carbon blacks (further reduced by the dilution line).

The following timepoints were chosen to collect the headspace samples: 0 h (T0), 2 h min (T1), 3,5 h (T2), 5 h (T3), 6,5 h (T4), 8 h (T5), and 24 h (T6), referring to bacteria inoculation into a pure TSB medium. After each gas sampling, 500 µL of the bacterial suspension was taken to assess bacterial growth by determining the colony-forming units (CFU/mL). Every single suspension was further diluted (from 10^−4^ to 10^−8^) in TSB, and 100 µL of the final dilution was plated on MacConkey agar and incubated at 37 °C for 24 h. Afterward, the colonies were counted to quantify the bacteria density/load in the samples.

In subsequent experiments, the impact of β-lactam antibiotic (imipenem, IMI) was investigated using both susceptible and resistant *E. coli* reference strains. A solution of imipenem in PBS at a concentration of 2.5 mg/L was prepared, and 0.5 mL of this solution was added to each of the four bottles containing bacterial cultures at timepoint T2 (3.5 h) immediately after sampling the headspace gas and bacterial suspension. The chosen concentration of 2.5 mg/L (equivalent to 2.5 µg/mL) was based on both clinical relevance and the relationship with the minimum inhibitory concentration (MIC) of *E. coli* established as ≤2 mg/L for susceptible and >4 mg/L for resistant strains, according to EUCAST.

### 4.3. Thermal Desorption–Gas Chromatography–Mass Spectrometry (TD-GC-MS)

Thermal desorption of VOCs trapped in the sorption tubes was carried out using a TD-30R autosampler (Shimadzu, Shim-Pol, Warsaw, Poland) coupled to a GC-MS system. Helium 6.0, purified using an HP-2 Heated Helium Purifier (ViCi Valco Instruments, Houston, TX, USA) and a dual set of Carrier Gas Purifiers (Agilent, Santa Clara, CA, USA), served as the carrier gas for thermal desorption at a flow rate of 60 mL/min. Sample desorption was performed at 320 °C for 15 min. The released analytes were focused on a cold trap at −20 °C and packed with a Carboxen adsorbent. Subsequently, the trapped compounds were injected into a Nexis GC-2030 gas chromatograph (Shimadzu, Shim-Pol, Warsaw, Poland) at 350 °C over 2 min in splitless mode, using a carrier gas flow rate of 0.82 mL/min (corresponding to a linear velocity of 33.0 cm/s). Chromatographic separation was achieved using an Rt-Q-Bond capillary column (30 m × 0.25 mm × 8 µm; Restek, Bellefonte, PA, USA) under the following temperature program: initial temperature of 60 °C held for 2 min, followed by a ramp of 8 °C/min to 110 °C (1 min hold), then 3 °C/min to 120 °C (7 min hold), 3 °C/min to 155 °C (7 min hold), 3°C/min to 225 °C (4 min hold), and finally 10 °C/min to 300 °C with a 7 min hold. Mass spectrometric detection was performed using a QP-2020 NX mass spectrometer (Shimadzu, Shim-Pol, Warsaw, Poland), operating in SCAN mode over a mass range of 33–235 *m*/*z*.

### 4.4. Data Processing and Statistical Analysis

The detected compounds were initially identified by comparing the acquired mass spectra with entries in the NIST 2017 Mass Spectra Library (Gaithersburg, MD, USA). Identification was further verified by comparing the retention times with those of measured standards. All reference substances used for metabolite identification were obtained from Alchem (Alchem, Toruń, Poland). Chromatographic peaks were first integrated using a customized method within the Shimadzu GC-MS PostRun Analysis software (version 4.45 SP1). Manual corrections were subsequently applied, where needed, by an experienced GC-MS analyst, whereby the “Target Ion” was assigned to each analyte as the most selective ion (unique for only one compound in the case of coeluted peaks) or the most abundant ion (for peaks resolved to the baseline). To verify peak identity and prevent errors in the integration of peak areas, the characteristic “reference ions” were also assigned to each analyte in PostRun Analysis software (Shimadzu, Shim-Pol, Warsaw, Poland). To evaluate the statistical significance of differences in VOC levels (expressed as peak areas of the corresponding chromatographic peaks) between bacterial suspensions at various growth stages and the reference TSB medium, the Kruskal–Wallis test was applied using Statistica 13.3 PL software (TIBCO Software Inc., Tulsa, OK, USA). This non-parametric test was chosen due to its robustness against outliers and its applicability to data that do not follow a normal distribution. A *p*-value below 0.05 was considered statistically significant. The Mann–Whitney U test was employed for pairwise comparisons of VOC abundance between two specific groups at a given timepoint. The false discovery rate (FDR) correction was also applied to account for multiple comparisons using Statistica 13.3. Significant VOCs identified through these tests had their peak areas log-transformed and visualized using both Statistica 13.3 PL and the online platform MetaboAnalyst 6.0 [56]. When necessary, missing data points, such as undetected VOCs, were handled using MetaboAnalyst software’s default imputation method, which replaces missing values with a small constant (typically half of the minimum positive value detected in the dataset).

## 5. Conclusions

In this study, we provide comprehensive insight into the kinetics of VOC release from *E. coli* under in vitro conditions, both in the absence and presence of imipenem, for susceptible and resistant strains. Three different patterns of VOC metabolism were identified: (1) continuous release correlating with overall bacterial load, (2) release with a temporary maximum in the period of highest bacterial growth activity, and (3) uptake of compounds, most likely utilized as a source of nutrients or energy. Dimethyl disulfide, 2-butenal, ethyl acetate, and furan were released significantly more strongly by the resistant strain compared to the susceptible one, suggesting their potential as markers of antimicrobial resistance. The addition of imipenem led to a significant reduction in the levels of many compounds in the susceptible strain, including 2-methyl-2-butene, Z-(2)-pentene, E-(2)-pentene, 1-butanol, n-propyl propionate, n-butyl acetate, and ethyl n-octanoate. Notably, these seven compounds were released by the resistant strain at comparable levels, both in the presence and absence of imipenem. The observation that only two-thirds of the VOCs significant for reference strains were also relevant for clinical strains suggests that further in vitro studies should focus on analyzing VOCs derived from clinical isolates. Although the range of VOCs emitted by clinical isolates is broader (which naturally hinders statistical comparison), this approach is more likely to have greater applicability and relevance for breath analysis in patients with VAP.

To achieve the distant future goal of an entirely non-invasive and rapid diagnostic of VAP along with pathogen identification, further in vitro studies on isolates of clinically relevant bacteria should be carried out. This would enable the identification of a distinct panel of metabolites associated explicitly with individual pathogenic species. Future studies should compare VOCs emitted by specific pathogenic bacteria under both in vitro and in vivo conditions to identify the most reliable biomarkers and evaluate their feasibility for timely and accurate point-of-care testing for respiratory infections [31]. The clinical relevance of the selected putative markers needs to be further confirmed in a large-scale multicenter clinical study.

## Figures and Tables

**Table 1:** The proliferation rate of the investigated cultures of *E. coli* cultures.

Timepoint	Mean CFU/mL for Susceptible Strain	Standard Deviation [CFU/mL] for Susceptible Strain	Mean CFU/mL for Resistant Strain	Standard Deviation [CFU/mL] for Resistant Strain	Mean CFU/mL for Isolates	Standard Deviation [CFU/mL] for Isolates
T0 (0 h)	0	0	0	0	0	0
T1 (2 h)	3.18 × 10^6^	2.15 × 10^6^	3.81 × 10^7^	1.35 × 10^7^	1.86 × 10^7^	1.16 × 10^7^
T2 (3.5 h)	1.08 × 10^8^	7.59 × 10^7^	4.35 × 10^8^	6.61 × 10^7^	2.77 × 10^8^	1.48 × 10^8^
T3 (5 h)	7.65 × 10^8^	4.42 × 10^8^	1.31 × 10^9^	9.15 × 10^7^	1.33 × 10^9^	4.19 × 10^8^
T4 (6.5 h)	9.15 × 10^8^	4.58 × 10^8^	1.52 × 10^9^	7.18 × 10^7^	1.67 × 10^9^	5.15 × 10^8^
T5 (8 h)	9.90 × 10^8^	4.91 × 10^8^	1.53 × 10^9^	2.05 × 10^8^	1.83 × 10^9^	5.74 × 10^8^
T6 (24 h)	1.61 × 10^9^	8.20 × 10^8^	1.67 × 10^9^	3.23 × 10^8^	2.44 × 10^9^	8.17 × 10^8^

**Table 2:** VOCs with the release profile directly proportional to the bacterial load. The values presented are mean peak area (n = 6 for susceptible *E. coli* and n = 5 for resistant strain).

Class	VOC	CAS Number	RT (min)	Strain	T0 (0 h)	T1 (2 h)	T2 (3.5 h)	T3 (5 h)	T4 (6.5 h)	T5 (8 h)	T6 (24 h)	Previously Reported
HC	Pentane (-n)	109-66-0	25,545	Susc.	1.46 × 10^4^	1.19 × 10^4^	1.34 × 10^4^	1.82 × 10^4^	*** 3.82 × 10^4^ ***	3.34 × 10^4^	2.68 × 10^4^	
				Res.	1.22 × 10^4^	1.18 × 10^4^	1.21× 10^4^	2.02 × 10^4^	* 2.24 × 10^4^ *	2.47 × 10^4^	2.65 × 10^4^	
	1-Hexene	592-41-6	36,856	Susc.	4.61 × 10^5^	7.34 × 10^5^	1.75 × 10^6^	3.65 × 10^6^	4.64 × 10^6^	**5.30 × 10^6^**	**6.57 × 10^6^**	
				Res.	3.86 × 10^3^	1.01 × 10^4^	8.60 × 10^3^	1.45 × 10^4^	1.68 × 10^4^	1.54 × 10^4^	1.50 × 10^4^	
	Heptane (-n)	142-82-5	51,257	Susc.	8.98 × 10^4^	8.55 × 10^4^	1.82 × 10^5^	9.21 × 10^5^	**1.36 × 10^6^**	**1.21 × 10^6^**	9.59 × 10^5^	
				Res.	1.06 × 10^5^	1.18 × 10^5^	3.03 × 10^5^	6.42 × 10^5^	7.74× 10^5^	**8.68 × 10^5^**	6.35 × 10^5^	
	Nonane (-n)	111-84-2	67,066	Susc.	1.87 × 10^5^	1.26 × 10^5^	6.92 × 10^5^	* 5.67 × 10^6^ *	*** 9.35 × 10^6^ ***	**9.17 × 10^6^**	3.62 × 10^6^	
				Res.	2.44 × 10^4^	1.69 × 10^5^	6.59 × 10^5^	* 2.19 × 10^6^ *	*** 3.10 × 10^6^ ***	**3.94 × 10^6^**	2.84 × 10^6^	
	1-Decene	872-05-9	71,200	Susc.	4.38 × 10^4^	5.14 × 10^4^	1.00 × 10^5^	* 5.08 × 10^5^ *	*** 9.41 × 10^5^ ***	**9.64 × 10^5^**	4.69 × 10^5^	
				Res.	5.27 × 10^4^	1.69 × 10^4^	7.02 × 10^4^	* 2.15 × 10^5^ *	* 3.36 × 10^6^ *	**4.53 × 10^5^**	**4.33 × 10^5^**	
	1-Undecene	821-95-4	72,300	Susc.	2.89 × 10^4^	2.14 × 10^4^	4.00 × 10^5^	*4.63 *× 10^6^	*** 8.48 × 10^6^ ***	**7.66 × 10^6^**	* 5.02 × 10^6^ *	
				Res.	4.41 × 10^4^	0	4.15 × 10^5^	* 2.42 × 10^6^ *	*** 3.53 × 10^6^ ***	**4.40 × 10^6^**	* 2.67 × 10^6^ *	
	Undecane (-n)	1120-21-4	73,000	Susc.	1.82 × 10^5^	1.35 × 10^5^	1.67 × 10^5^	* 4.99× 10^5^ *	* 8.23 **×** 10^5^ *	8.19 × 10^5^	**1.37 × 10^6^**	
				Res.	1.67 × 10^5^	5.80 × 10^4^	7.30 × 10^4^	* 1.88 × 10^6^ *	* 3.06 × 10^5^ *	3.68 × 10^5^	7.72 × 10^5^	
alc.	Ethanol	64-17-5	14,000	Susc.	1.32 × 10^7^	1.39 × 10^7^	1.93 × 10^8^	4.89 × 10^8^	**5.49 × 10^8^**	5.12 × 10^8^	**6.40 × 10^8^**	[5,8,11,12,13,15,16,21,22,23,24]
				Res.	1.37 × 10^7^	1.09 × 10^7^	2.54 × 10^8^	5.01 × 10^8^	**5.46 × 10^8^**	5.46 × 10^8^	**6.02 × 10^8^**	
	1-Propanol	71-23-8	26,439	Susc.	1.23 × 10^6^	1.50 × 10^6^	1.40 × 10^7^	* 5.45 × 10^7^ *	*** 1.29 × 10^8^ ***	*** 1.25 × 10^8^ ***	*** 1.16 × 10^8^ ***	[3,5,16,21,22]
				Res.	9.95 × 10^5^	1.28 × 10^6^	9.43 × 10^6^	* 1.82 × 10^7^ *	*** 2.03 × 10^7^ ***	*** 2.49 × 10^7^ ***	* 1.53 × 10^7^ *	
	2-Methyl-1-Propanol	78-83-1	36,856	Susc.	8.39 × 10^5^	1.18 × 10^6^	2.93 × 10^6^	6.34 × 10^6^	**7.88 × 10^6^**	**7.03 × 10^6^**	**9.02 × 10^6^**	[5,22]
				Res.	1.33 × 10^6^	1.26 × 10^6^	3.90 × 10^6^	6.91 × 10^6^	7.66 × 10^6^	**8.45 × 10^6^**	7.88 × 10^6^	
	1-Butanol	71-363	40,196	Susc.	1.94 × 10^6^	1.04 × 10^6^	1.50 × 10^6^	5.74 × 10^6^	* 3.27 × 10^7^ *	3.66 × 10^7^	**4.52 × 10^7^**	[5,9,11,14,15,22,25]
				Res.	9.97 × 10^5^	8.69 × 10^5^	1.76 × 10^6^	7.56 × 10^6^	* 2.26 × 10^7^ *	**3.05 × 10^7^**	**2.85 × 10^7^**	
	3-Methyl-1-Butanol	123-51-3	50,395	Susc.	8.29 × 10^5^	2.25 × 10^6^	1.27 × 10^7^	**2.68 × 10^7^**	**3.00 × 10^7^**	**2.61 × 10^7^**	**3.26 × 10^7^**	[10,11,25]
				Res.	1.17 × 10^6^	3.67 × 10^6^	1.75 × 10^7^	2.86 × 10^7^	**3.04 × 10^7^**	**3.17 × 10^7^**	**2.82 × 10^7^**	
ald.	Acetaldehyde	75-07-0	9644	Susc.	1.32 × 10^7^	1.10 × 10^7^	3.41 × 10^7^	**7.19 × 10^7^**	5.64 × 10^7^	4.78 × 10^7^	*** 8.61 × 10^7^ ***	[11,12,16,21,25]
				Res.	1.12 × 10^7^	9.71 × 10^6^	4.63 × 10^7^	**7.36 × 10^7^**	4.99 × 10^7^	5.05 × 10^7^	* 5.55 × 10^7^ *	
	Octanal	124-13-0	70.320	Susc.	3.83 × 10^4^	2.89 × 10^4^	1.04 × 10^5^	* 4.86 × 10^5^ *	*** 6.11 × 10^5^ ***	**5.49 × 10^5^**	4.31 × 10^5^	[23]
				Res.	4.67 × 10^4^	2.85 × 10^4^	1.09 × 10^5^	* 2.79 × 10^5^ *	* 3.22 × 10^5^ *	**3.84 × 10^5^**	2.51× 10^5^	
	Decanal	112-31-2	76,452	Susc.	2.16 × 10^4^	3.88 × 10^4^	2.80 × 10^5^	*** 1.35 × 10^6^ ***	*** 1.87 × 10^6^ ***	**1.90 × 10^6^**	8.04 × 10^5^	[14,23]
				Res.	1.52 × 10^5^	3.33 × 10^4^	2.44 × 10^5^	* 5.97 × 10^5^ *	* 7.35 × 10^5^ *	**1.07 × 10^6^**	8.21 × 10^5^	
ket.	2-Pentanone	107-87-9	44,911	Susc.	6.88 × 10^5^	8.41 × 10^5^	1.34 × 10^6^	1.33 × 10^6^	1.17 × 10^6^	1.17× 10^6^	**1.46 × 10^6^**	[26]
				Res.	5.90 × 10^5^	1.15 × 10^6^	1.64 × 10^6^	1.48 × 10^6^	1.20 × 10^6^	1.32× 10^6^	1.97 × 10^6^	
	2-Heptanone	110-43-0	63,501	Susc.	6.83 × 10^4^	8.30 × 10^4^	8.67 × 10^4^	8.52 × 10^4^	1.48 × 10^5^	1.61× 10^5^	**2.90 × 10^5^**	[3,4,7,14,22,27]
				Res.	9.16 × 10^4^	8.76 × 10^4^	6.38 × 10^4^	1.18 × 10^5^	1.54 × 10^5^	1.79× 10^5^	5.93 × 10^5^	
esters	Ethyl Acetate	141-78-6	33,382	Susc.	1.54 × 10^6^	6.95 × 10^5^	3.03× 10^6^	1.96 × 10^7^	4.04 × 10^7^	*** 4.19 × 10^7^ ***	**9.22 × 10^7^**	[15,22]
				Res.	1.04 × 10^6^	6.51 × 10^5^	4.26× 10^6^	2.66 × 10^7^	3.97 × 10^7^	*** 5.61 × 10^7^ ***	**8.18 × 10^7^**	
	Ethyl Propionate	105-37-3	46,658	Susc.	0	0	0	1.68 × 10^4^	* 1.50 × 10^6^ *	* 2.61 × 10^6^ *	*** 7.78 × 10^6^ ***	
				Res.	0	0	0	0	* 1.47 × 10^4^ *	* 2.33 × 10^5^ *	*** 3.53 × 10^6^ ***	
	Ethyl Butyrate	105-54-4	55,760	Susc.	0	0	0	1.66 × 10^4^	7.38 × 10^4^	6.11 × 10^4^	**3.80 × 10^5^**	[7,15]
				Res.	0	0	0	1.39 × 10^4^	5.53 × 10^4^	8.33 × 10^4^	**2.70 × 10^5^**	
	n-Propyl Propionate	106-36-5	56,260	Susc.	0	0	0	0	9.23 × 10^4^	* 3.30 × 10^5^ *	*** 9.96 × 10^5^ ***	
				Res.	0	0	0	0	0	* 0 *	* 4.78 × 10^4^ *	
	n-Butyl Acetate	123-86-4	56,755	Susc.	3.33 × 10^4^	9.19 × 10^3^	1.00 × 10^4^	8.86 × 10^4^	2.27 × 10^5^	2.67 × 10^5^	**4.58 × 10^5^**	[22]
				Res.	1.29 × 10^4^	9.51 × 10^3^	1.62 × 10^4^	9.45 × 10^4^	2.07 × 10^5^	2.69 × 10^5^	4.17 × 10^5^	
	3-Methylbutyl Acetate	123-92-2	62,865	Susc.	5.00 × 10^3^	1.00 × 10^4^	3.17 × 10^4^	**7.49 × 10^4^**	**8.50 × 10^4^**	6.65 × 10^4^	**1.05 × 10^5^**	
				Res.	3.19 × 10^3^	8.63 × 10^3^	4.30 × 10^4^	8.76 × 10^4^	9.43 × 10^4^	9.82 × 10^4^	1.18 × 10^5^	
	n-Octyl acetate	112-14-1	75,906	Susc.	0	2.28 × 10^4^	9.50 × 10^4^	4.41 × 10^5^	*** 7.51 × 10^5^ ***	**6.24 × 10^5^**	3.60 × 10^5^	
				Res.	0	0	3.42 × 10^4^	3.47 × 10^5^	*** 4.05 × 10^5^ ***	**4.85 × 10^5^**	2.79 × 10^5^	
VSCs	Methanethiol	74-93-1	10,900	Susc.	3.44 × 10^6^	3.41 × 10^6^	5.27 × 10^6^	**1.27 × 10^7^**	**1.53 × 10^7^**	**1.38 × 10^7^**	1.03 × 10^7^	[11,12,16,22]
				Res.	2.26 × 10^6^	3.42 × 10^6^	6.14 × 10^6^	**1.55 × 10^7^**	**1.66 × 10^7^**	**1.63 × 10^7^**	7.79 × 10^6^	
	Mercaptoacetone	24653-75-6	41,956	Susc.	1.24 × 10^5^	7.37 × 10^4^	9.82 × 10^4^	2.88 × 10^5^	**5.82 × 10^5^**	4.42 × 10^5^	4.21 × 10^5^	
				Res.	1.05 × 10^5^	6.47 × 10^4^	7.69 × 10^4^	3.71 × 10^5^	4.67 × 10^5^	5.15 × 10^5^	4.14 × 10^5^	
	Dimethyl Disulfide (DMDS)	624-92-0	47,136	Susc.	2.85 × 10^7^	2.30 × 10^7^	2.87 × 10^7^	6.33 × 10^7^	7.40 × 10^7^	* 5.64 × 10^7^ *	**9.72 × 10^7^**	[5,6,14,16,23]
				Res.	2.14 × 10^7^	2.19 × 10^7^	4.62 × 10^7^	8.25 × 10^7^	9.73 × 10^7^	*** 1.10 × 10^8^ ***	5.98 × 10^7^	
other	Ethyl Vinyl Ether	109-92-2	23,548	Susc.	6.55 × 10^3^	5.67 × 10^3^	9.35 × 10^3^	**8.82 × 10^4^**	7.05 × 10^4^	4.01 × 10^5^	**3.94 × 10^5^**	
				Res.	8.46 × 10^3^	5.40 × 10^3^	1.80 × 10^4^	**9.43 × 10^4^**	5.74 × 10^4^	5.46 × 10^4^	**2.62 × 10^5^**	
	Indole	120-72-9	77,470	Susc.	9.13 × 10^5^	4.42 × 10^5^	4.56 × 10^5^	4.68 × 10^5^	* 1.62 × 10^6^ *	* 1.08 × 10^7^ *	*** 1.66 × 10^8^ ***	[3,4,5,6,7,8,9,10,11,12,13,14,15,16,17]
				Res.	6.41 × 10^5^	3.18 × 10^5^	2.46 × 10^5^	3.25 × 10^5^	* 3.43 × 10^5^ *	* 6.41× 10^5^ *	* 1.17 × 10^8^ *	

Table legend: VOCs are volatile organic compounds. CAS stands for Chemical Abstract Service, providing a unique number that identifies a substance. RT—retention time, Susc.—a susceptible strain of *E. coli*, Res.—a resistant strain of *E. coli*, HC—hydrocarbons, alc.—alcohol, ald.—aldehydes, ket.—ketones, VSCs—volatile sulfuric compounds. T0–T6 refer to sampling points at different times of bacterial growth (see Section 3.1 for more details). Bold value of peak area indicates the statistically significant difference (Kruskal–Wallis test) between the timepoint of interest and T0. Underlined italic value of peak area in blue font indicates the statistically significant difference (Mann–Whitney U test) between resistant and susceptible strains at the same timepoint.

**Table 3:** VOCs with a temporary maximum release profile. The values presented are mean peak area (n = 6 for susceptible *E. coli* and n = 5 for resistant strain).

Class	VOC	CAS Number	RT (min)	Strain	T0 (0 h)	T1 (2 h)	T2 (3.5 h)	T3 (5 h)	T4 (6.5 h)	T5 (8 h)	T6 (24 h)	Previously Reported
HC	1,3-Butadiene	106-99-0	12,987	Susc.	2.85 × 10^5^	2.34× 10^5^	4.72 × 10^5^	**2.10 × 10^6^**	* 1.34 × 10^6^ *	1.05 × 10^6^	1.74 × 10^6^	
				Res.	1.45 × 10^5^	2.45 × 10^5^	7.64 × 10^5^	**2.08 × 10^6^**	* 8.94 × 10^5^ *	9.86 × 10^5^	1.08 × 10^6^	
	2-Methyl-2-Butene	513-35-9	21,456	Susc.	2.86 × 10^5^	2.86 × 10^5^	3.87 × 10^5^	2.41 × 10^6^	**4.78 × 10^6^**	3.08 × 10^6^	2.28 × 10^5^	
				Res.	2.83 × 10^5^	2.89 × 10^5^	3.30 × 10^5^	2.30 × 10^6^	**4.48 × 10^6^**	**3.63 × 10^6^**	4.81 × 10^5^	
	Isoprene	78-79-5	24,927	Susc.	4.24× 10^5^	4.82 × 10^5^	2.45 × 10^6^	**1.08 × 10^7^**	**7.64 × 10^6^**	3.58 × 10^6^	1.36 × 10^6^	[3,9,15,21]
				Res.	3.85× 10^5^	6.01 × 10^5^	3.62 × 10^6^	**9.62 × 10^6^**	**6.47 × 10^6^**	5.26 × 10^6^	8.54 × 10^5^	
	2-Pentene (Z-)	627-20-3	25,271	Susc.	1.33× 10^5^	1.10 × 10^5^	3.21 × 10^5^	5.08 × 10^6^	**1.27 × 10^7^**	**8.37 × 10^6^**	3.56 × 10^5^	
				Res.	2.15× 10^5^	1.43 × 10^5^	2.38 × 10^5^	5.35 × 10^6^	**1.22 × 10^7^**	9.61 × 10^6^	3.42 × 10^5^	
	2-Pentene (E-)	627-20-3	25,680	Susc.	1.34× 10^5^	1.09 × 10^5^	3.18 × 10^5^	5.11 × 10^6^	**1.27 × 10^7^**	**8.37 × 10^6^**	3.63 × 10^5^	
				Res.	2.08× 10^5^	1.51 × 10^5^	2.25 × 10^5^	5.37 × 10^6^	**1.22 × 10^7^**	9.63 × 10^6^	3.38 × 10^5^	
	(E)-1,3-Pentadiene	2004-70-8	27,082	Susc.	8.02 × 10^4^	6.42 × 10^4^	1.02 × 10^5^	**2.33 × 10^5^**	* 1.93 × 10^5^ *	1.58 × 10^5^	2.14 × 10^5^	
				Res.	8.35 × 10^4^	7.47 × 10^4^	1.34 × 10^5^	2.20 × 10^5^	* 1.17 × 10^5^ *	1.23 × 10^5^	1.84 × 10^5^	
ald.	Propanal	123-38-6	18,263	Susc.	4.16 × 10^5^	5.91 × 10^5^	2.58 × 10^6^	*** 4.01 × 10^6^ ***	*** 1.29 × 10^7^ ***	* 3.59 × 10^6^ *	* 2.96 × 10^6^ *	[25]
				Res.	2.75 × 10^5^	6.16 × 10^5^	**1.40 × 10^6^**	*** 1.33 × 10^6^ ***	* 8.32 × 10^5^ *	* 1.05 × 10^6^ *	* 4.55 × 10^5^ *	
	Butanal	123-72-8	31,169	Susc.	1.35 × 10^6^	1.47 × 10^6^	1.66 × 10^6^	**4.69 × 10^6^**	**3.71 × 10^6^**	3.03 × 10^6^	3.24 × 10^6^	[9,22]
				Res.	8.05 × 10^5^	1.58 × 10^6^	2.29 × 10^6^	**4.84 × 10^6^**	2.70 × 10^6^	2.97 × 10^6^	2.97 × 10^6^	
	2-Butenal	4170-30-3	35,863	Susc.	1.77 × 10^6^	2.18 × 10^6^	* 5.81 × 10^6^ *	**2.23 × 10^7^**	* 5.57× 10^6^ *	2.53 × 10^6^	9.09 × 10^6^	
				Res.	5.33 × 10^5^	2.48 × 10^6^	*** 1.21 × 10^7^ ***	**2.35 × 10^7^**	* 2.81 × 10^6^ *	2.92 × 10^6^	5.18 × 10^6^	
esters	Ethyl n-Octanoate	106-32-1	75,589	Susc.	0	0	0	9.42 × 10^5^	*** 3.56 × 10^6^ ***	**2.54 × 10^6^**	8.26 × 10^4^	
				Res.	0	0	0	5.84 × 10^5^	*** 1.25 × 10^6^ ***	**1.44 × 10^6^**	2.25 × 10^5^	
ket.	Acetone	67-64-1	18,266	Susc.	1.37× 10^8^	1.63 × 10^8^	1.67 × 10^8^	1.76 × 10^8^	1.74 × 10^8^	1.61 × 10^8^	1.41 × 10^8^	[5,11,15,17,23,25,26]
				Res.	7.89 × 10^7^	1.61 × 10^8^	1.39 × 10^8^	**1.73 × 10^8^**	1.70 × 10^8^	1.69 × 10^8^	1.10 × 10^8^	
	3-Pentene-2-one, (E)	625-33-2	48,521	Susc.	1.80 × 10^5^	6.15 × 10^5^	**9.50 × 10^5^**	5.23 × 10^5^	1.88 × 10^5^	1.61 × 10^5^	3.53× 10^5^	
				Res.	1.12 × 10^5^	**1.12 × 10^6^**	7.78 × 10^5^	5.33 × 10^5^	1.49 × 10^5^	1.66 × 10^5^	2.39 × 10^5^	
VSCs	Carbon Disulfide	75-15-0	20,163	Susc.	9.09 × 10^5^	9.43 × 10^5^	9.97 × 10^5^	1.30 × 10^6^	2.24 × 10^6^	1.14 × 10^6^	7.18 × 10^5^	[9,21]
				Res.	4.44 × 10^5^	9.88 × 10^5^	1.09E + 06	1.54 × 10^6^	**2.57 × 10^6^**	1.40 × 10^6^	4.93 × 10^5^	
	Dimethyl Sulfide	75-18-3	20,432	Susc.	7.96 × 10^5^	3.91 × 10^5^	3.44 × 10^6^	**2.75 × 10^7^**	**1.48 × 10^7^**	8.51 × 10^6^	4.31 × 10^6^	[15,21,23,26]
				Res.	5.12 × 10^5^	2.49 × 10^5^	1.97× 10^6^	**2.62 × 10^7^**	1.56 × 10^7^	1.03 × 10^7^	3.19 × 10^6^	
	Ethyl Methyl Sulfide	624-89-5	32,634	Susc.	0	0	6.89 × 10^4^	**1.19 × 10^6^**	**1.17 × 10^6^**	**6.51** × 10^5^	6.59 × 10^4^	[23]
				Res.	0	0	5.21 × 10^4^	**9.06 × 10^5^**	**1.06 × 10^6^**	8.07 × 10^5^	3.29 × 10^4^	
	Dimethyl sulfone	67-71-0	59,629	Susc.	3.35× 10^5^	0	5.69 × 10^5^	1.49 × 10^6^	**2.15 × 10^6^**	1.54 × 10^6^	5.90 × 10^5^	
				Res.	2.85× 10^5^	1.00 × 10^4^	3.47 × 10^5^	1.31 × 10^6^	1.69 × 10^6^	1.4 × 10^6^	3.29 × 10^5^	
other	Furan	110-00-9	17,916	Susc.	6.19× 10^5^	6.92 × 10^5^	*1.16 *× 10^6^	**4.17 × 10^6^**	**1.83 × 10^6^**	1.27 × 10^6^	1.56 × 10^6^	
				Res.	3.56 × 10^5^	6.07 × 10^5^	*2.21 *× 10^6^	**4.15 × 10^6^**	1.41 × 10^6^	1.39 × 10^6^	1.21 × 10^6^	

Table legend: VOCs are volatile organic compounds. CAS stands for Chemical Abstract Service, providing a unique number that identifies a substance. RT—retention time, Susc.—a susceptible strain of *E. coli*, Res.—a resistant strain of *E. coli*, HC—hydrocarbons, ald.—aldehydes, ket.—ketones, VSCs—volatile sulfuric compounds. T0-T6 refer to sampling points at different times of bacterial growth (see Section 3.1. for more details). Bold value of peak area indicates the statistically significant difference (Kruskal–Wallis test) between the timepoint of interest and T0. Underlined italic value of peak area in blue font indicates the statistically significant difference (Mann–Whitney U test) between resistant and susceptible strains at the same timepoint.

**Table 4:** VOCs taken up by *E. coli* during the course of the experiment. The values presented are mean peak area (n = 6 for susceptible *E. coli* and n = 5 for resistant strain).

Class	VOC	CAS Number	RT (min)	Strain	T0 (0 h)	T1 (2 h)	T2 (3.5 h)	T3 (5 h)	T4 (6.5 h)	T5 (8 h)	T6 (24 h)	Previously Reported
HC	iso-Butane	75-28-5	12,000	Susc.	6.06 × 10^6^	5.59 × 10^6^	* 3.15 × 10^6^ *	**8.76 × 10^5^**	**8.38 × 10^5^**	**8.16 × 10^5^**	**5.97 × 10^5^**	
				Res.	1.93 × 10^6^	4.87 × 10^6^	* 1.04 × 10^6^ *	7.93 × 10^5^	8.76 × 10^5^	9.65× 10^5^	8.43 × 10^5^	
	2-methyl-1-propene	115-11-7	12,629	Susc.	6.13 × 10^6^	5.48 × 10^6^	* 3.22 × 10^6^ *	1.11 × 10^6^	**9.51 × 10^5^**	**8.68 × 10^5^**	**6.42 × 10^5^**	
				Res.	2.27 × 10^6^	4.62 × 10^6^	* 1.27 × 10^6^ *	1.01 × 10^6^	9.08 × 10^5^	8.65 × 10^5^	8.02 × 10^5^	
	(Z)-2-Butene	590-18-1	12,766	Susc.	2.99 × 10^6^	2.49 × 10^6^	* 1.73 × 10^6^ *	**1.20 × 10^6^**	**1.23 × 10^6^**	**1.13 × 10^6^**	1.33 × 10^6^	
				Res.	1.61× 10^6^	2.15 × 10^6^	* 1.12 × 10^6^ *	1.03 × 10^6^	9.53× 10^5^	9.68× 10^5^	1.09 × 10^6^	
	n-Butane	106-97-8	13,378	Susc.	7.88 × 10^4^	7.31 × 10^4^	6.73 × 10^4^	3.25× 10^4^	3.44 × 10^4^	3.92 × 10^4^	**2.59 × 10^4^**	
				Res.	4.14 × 10^4^	6.92 × 10^4^	5.58 × 10^4^	3.06 × 10^4^	3.72 × 10^4^	4.12× 10^4^	2.60 × 10^4^	
	(E)-2-Butene	624-64-6	13,659	Susc.	4.88 × 10^5^	4.52 × 10^5^	3.25 × 10^5^	1.16 × 10^5^	**6.00 × 10^4^**	**6.16 × 10^4^**	**6.38 × 10^4^**	
				Res.	2.41 × 10^5^	4.16 × 10^5^	2.07 × 10^5^	9.21 × 10^4^	6.49 × 10^4^	6.54 × 10^4^	6.07 × 10^4^	
ald.	Methacrolein	78-85-3	28,261	Susc.	1.27 × 10^7^	1.20 × 10^7^	9.42 × 10^6^	* 2.73 × 10^6^ *	**9.86 × 10^5^**	**4.46 × 10^5^**	**6.73 × 10^5^**	
				Res.	9.08 × 10^6^	1.21 × 10^7^	8.46 × 10^6^	* 1.48 × 10^6^ *	7.11 × 10^5^	**4.76 × 10^5^**	**4.55 × 10^5^**	
	2-Methylpropanal	78-84-2	28,588	Susc.	1.54 × 10^7^	1.95 × 10^7^	* 1.73 × 10^7^ *	* 3.34 × 10^6^ *	5.90 × 10^5^	**2.84 × 10^5^**	**2.19 × 10^5^**	[9,22]
				Res.	8.49 × 10^6^	1.68 × 10^7^	* 7.18 × 10^6^ *	* 9.87× 10^5^ *	3.86 × 10^5^	2.54 × 10^5^	**1.63 × 10^5^**	
	2-Ethylacrolein	922-63-4	41,552	Susc.	3.32 × 10^6^	2.61 × 10^6^	1.54 × 10^6^	2.65 × 10^5^	**7.47 × 10^4^**	**5.16 × 10^4^**	**1.96 × 10^5^**	
				Res.	3.38 × 10^6^	2.29 × 10^6^	9.65 × 10^5^	1.68 × 10^5^	**5.81 × 10^4^**	**8.15 × 10^4^**	1.31× 10^5^	
	3-MethylButanal	590-86-3	42,417	Susc.	1.53 × 10^6^	1.33 × 10^8^	* 7.18 × 10^7^ *	3.83 × 10^6^	**1.30 × 10^6^**	**1.04 × 10^6^**	**1.09 × 10^6^**	[3,4,5,6,9,22]
				Res.	1.09 × 10^8^	1.24 × 10^8^	* 1.61 × 10^7^ *	2.24 × 10^6^	1.35 × 10^6^	**1.19 × 10^6^**	**9.53 × 10^5^**	
	2-Methyl-2-Butenal	1115-11-3	47,964	Susc.	7.70 × 10^6^	7.01 × 10^6^	4.73 × 10^6^	1.68 × 10^6^	**1.10 × 10^6^**	**7.42 × 10^5^**	**3.52 × 10^5^**	
				Res.	5.65 × 10^6^	6.33 × 10^6^	2.76 × 10^6^	1.37 × 10^6^	9.95 × 10^5^	7.71 × 10^5^	**2.22 × 10^5^**	
	Furfural	98-01-1	53,615	Susc.	1.53 × 10^6^	9.14 × 10^5^	8.78 × 10^5^	7.16 × 10^5^	**3.49 × 10^5^**	6.79 × 10^5^	1.37× 10^6^	
				Res.	1.64 × 10^6^	9.70 × 10^5^	7.78 × 10^5^	7.60 × 10^5^	**4.83 × 10^5^**	5.90 × 10^5^	9.59× 10^5^	
	Benzaldehyde	100-52-7	65,584	Susc.	2.82 × 10^7^	2.84 × 10^7^	2.68 × 10^7^	* 1.38 × 10^7^ *	* 3.10 × 10^6^ *	**1.43 × 10^6^**	**2.61 × 10^6^**	[3,5,7,17,25]
				Res.	2.71 × 10^7^	2.90 × 10^7^	1.90 × 10^7^	* 2.34 × 10^6^ *	*** 1.26 × 10^6^ ***	**1.25 × 10^6^**	1.97 × 10^6^	
esters	Methyl Methacrylate	80-62-6	45,514	Susc.	2.16 × 10^6^	1.76 × 10^6^	1.75 × 10^6^	1.88 × 10^6^	1.77 × 10^6^	1.47 × 10^6^	**1.02 × 10^6^**	
				Res.	2.00 × 10^6^	2.15 × 10^6^	2.11 × 10^6^	2.52 × 10^6^	2.35 × 10^6^	2.23 × 10^6^	1.05 × 10^6^	

Table legend: VOCs—volatile organic compounds. CAS stands for Chemical Abstract Service, providing a unique number identifying a substance, RT—retention time, Susc.—a susceptible strain of *E. coli*, Res.—a resistant strain of *E. coli*, HC—hydrocarbons, ald.—aldehydes. T0-T6 refer to sampling points at different times of bacterial growth (see Section 3.1. for more details). Bold peak area value indicates the statistically significant difference (Kruskal–Wallis test) between the timepoint of interest and T0. Underlined italic value of peak area in blue font indicates the statistically significant difference (Mann–Whitney U test) between resistant and susceptible strains at the same timepoint.

**Table 5:** Statistical comparison of VOC concentrations at timepoint T5 (8 h) with and without imipenem (IMI) addition to the susceptible and resistant *Escherichia coli* cultures. The *p*-values were calculated with the Mann–Whitney U non-parametric test (with FDR correction) for VOC level at timepoint T5 (8 h of bacteria incubation) without and after imipenem addition, separately for sensitive and resistant strains. See table legends for more details.

	Original Profile	“*p*” for Susceptible	After IMI Addition	“*p*” for Resistant	After IMI Addition
Acetaldehyde	S ↑,R ↑	0.229767	no change	0.008114	R ↓
Methanethiol	S ↑,R ↑	0.005075	S ↓	0.008114	R ↓
iso-Butane	S ↓, N.S res.	0.810181	no change	0.022480	Res N.S.↓
2-methyl-1-propene	S ↓, N.S res.	0.471171	no change	0.008114	Res N.S.↓
(Z)-2-Butene	S ↓, N.S res.	0.030640	S ↓	0.008114	Res N.S.↓
1,3-Butadiene	S^TM^,R^TM^	0.128206	no change	0.008114	R ↓
n-Butane	S ↓, N.S res.	0.065553	no change	0.013711	Res N.S.↓
(E)-2-Butene	S ↓, R ↓	0.045328	S ↑	0.022480	R ↓
Ethanol	S ↑,R ↑	0.008239	S ↓	0.013711	R ↓
Furan	S^TM^,R^TM^	0.173486	no change	0.008114	R ↓
Propanal	S^TM^,R^TM^	0.045328	S ↓	0.013711	R ↓
Acetone	R↑, N.S. susc.	0.173486	no change	0.082838	no change
Carbon Disulfide	R^TM^, N.S. susc.	0.092697	no change	0.235334	no change
Dimethyl Sulfide	S^TM^,R^TM^	0.575174	no change	0.022480	R ↓
2-Methyl-2-Butene	S^TM^,R^TM^	0.005075	S ↓	0.522817	no change
Ethyl Vinyl Ether	S ↑,R ↑	0.810181	no change	0.055235	no change
Isoprene	S^TM^,R^TM^	0.297954	no change	0.008114	R ↓
Pentane (-n)	S ↑, N.S res.	0.013066	S ↓	0.082838	Res N.S.↓
2-Pentene (Z-)	S^TM^,R^TM^	0.005075	S ↓	0.927265	no change
2-Pentene (E-)	S^TM^,R^TM^	0.005075	S ↓	0.927265	no change
1-Propanol	S ↑,R ↑	0.005075	S ↓	0.170904	no change
(E)-1,3-Pentadiene	S^TM^, N.S res.	0.005075	S ↓	0.008114	Res N.S.↓
Methacrolein	S ↓, R ↓	0.005075	S ↑	0.035765	R ↓
2-Methylpropanal	S ↓, R ↓	0.005075	S ↑	0.170904	no change
Butanal	S^TM^,R^TM^	0.005075	S ↓	0.008114	R ↓
Ethyl Methyl Sulfide	S^TM^,R^TM^	0.065553	no change	0.022480	R ↓
Ethyl Acetate	S ↑,R ↑	0.013066	S ↓	0.035765	R ↓
2-Butenal	S^TM^,R^TM^	0.013066	S ↑	0.008114	R ↓
1-Hexene	S ↑, N.S res.	0.013066	S ↓	0.013711	Res N.S.↓
2-Methyl-1-Propanol	S ↑,R ↑	0.013066	S ↓	0.008114	R ↓
1-Butanol	S ↑,R ↑	0.005075	S ↓	0.055235	no change
2-Ethylacrolein	S ↓, R ↓	0.020241	S ↑	0.022480	R ↓
Mercaptoacetone	S ↑, N.S res.	0.173486	no change	0.035765	Res N.S.↓
3-MethylButanal	S ↓, R ↓	0.005075	S ↑	0.013711	R ↓
2-Pentanone	S ↑, N.S res.	0.013066	S ↓	0.008114	Res N.S.↓
Methyl Methacrylate	S ↓, N.S res.	0.936186	no change	0.120692	no change
Dimethyl Disulfide (DMDS)	S ↑,R ↑	0.013066	S ↓	0.008114	R ↓
Ethyl Propionate	S ↑,R ↑	0.004772	S ↓	0.008114	R ↓
2-Methyl-2-Butenal	S ↓, R ↓	0.173486	no change	0.082838	no change
3-Penten-2-one, (E)	S^TM^,R^TM^	0.297954	no change	0.315303	no change
3-Methyl-1-Butanol	S ↑,R ↑	0.173486	no change	0.120692	no change
Heptane (-n)	S ↑,R ↑	0.005075	S ↓	0.008114	R ↓
Furfural	S ↓, R ↓	0.688921	no change	0.022480	R ↓
Ethyl Butyrate	S ↑,R ↑	0.377643	no change	0.022480	R ↓
n-Propyl Propionate	S ↑, N.S res.	0.002779	S ↓	0.927265	no change
n-Butyl Acetate	S ↑, N.S res.	0.006151	S ↓	0.035765	Res N.S.↓
Dimethyl solfone	S^TM^, N.S res.	0.297954	no change	0.120692	no change
3-Methylbutyl Acetate (banana oil)	S ↑, N.S res.	0.128206	no change	0.055235	no change
2-Heptanone	S ↑, N.S res.	0.092697	no change	0.927265	no change
Benzaldehyde	S ↓, R ↓	0.005075	S ↑	0.008114	R ↓
Nonane (-n)	S ↑,R ↑	0.013066	S ↓	0.013711	R ↓
Octanal	S ↑,R ↑	0.008239	S ↓	0.008114	R ↓
1-Decene	S ↑,R ↑	0.020241	S ↓	0.008114	R ↓
1-Undecene	S ↑,R ↑	0.045328	S ↓	0.008114	R ↓
Undecane (-n)	S ↑, N.S res.	0.030640	S ↓	0.022480	Res N.S.↓
Ethyl n-Octanoate	S^TM^,R^TM^	0.005075	S ↓	0.120692	no change
n-Octyl acetate	S ↑,R ↑	0.005075	S ↓	0.008114	R ↓
Decanal	S ↑,R ↑	0.008239	S ↓	0.008114	R ↓
Indole	S ↑, N.S res.	0.005075	S ↓	0.008114	Res N.S.↓

Table legend: IMI = imipenem, ↑—release profile, ↓ = uptake profile, TM = temporary maximum profile, S = susceptible strain, R = resistant strain, N.S. res = compound was not significant for pure *E. coli* resistant strain, N.S. susc = compound was not significant for pure *E. coli* susceptible strain ↑ = increase in VOC amount after imipenem addition, ↓ = decrease in VOC amount after imipenem addition; “*p*” values marked in blue font indicate statistically significant changes in the amount of VOCs released.

**Table 6:** Statistically significant VOCs observed in the culture headspace of *E. coli* clinical isolates.

	Class	VOC	CAS	RT (min)	T0		T3		T5		T6		Previously Reported
					Mean Peak Area	Occur. (n = 21)	Mean Peak Area	Occur. (n = 21)	Mean Peak Area	Occur. (n = 21)	Mean Peak Area	Occur. (n = 16)	
RELEASE	HC	Propene	115-07-1	7250	3.42 × 10^6^	21	1.78 × 10^6^	21	**1.24 × 10^6^**	21	**5.80 × 10^5^**	15	[12]
		Pentane (-n)^C^	109-66-0	25,545	1.01 × 10^4^	21	1.42 × 10^4^	21	**1.82 × 10^4^**	21	1.29 × 10^4^	16	
		1-Hexene ^C^	592-41-6	36,856	4.07 × 10^5^	21	**1.72 × 10^6^**	21	*** 2.08 × 10^6^ ***	21	*** 1.86 × 10^6^ ***	16	
		Heptane (-n) ^C^	142-82-5	51,257	8.80 × 10^4^	21	**4.79 × 10^5^**	21	*** 5.82 × 10^5^ ***	21	**2.77 × 10^5^**	16	
		Nonane (-n) ^C^	111-84-2	67,066	2.15 × 10^5^	21	**1.83 × 10^6^**	21	*** 2.68 × 10^6^ ***	21	1.05 × 10^6^	16	
		1-Decene	872-05-9	71,200	5.01 × 10^4^	10	**2.51 × 10^5^**	20	*** 4.72 × 10^5^ ***	20	*** 4.43 × 10^5^ ***	14	
		1-Undecene	821-95-4	72,300	1.23 × 10^5^	3	**1.92 × 10^6^**	18	*** 2.19 × 10^6^ ***	21	7.50 × 10^5^	13	
		Undecane (-n)	1120-21-4	73,000	1.45 × 10^5^	11	**9.87 × 10^5^**	20	**9.62 × 10^5^**	21	*** 5.67 × 10^5^ ***	13	
	alc.	Ethanol ^C^	64-17-5	14,000	5.33 × 10^6^	21	**4.04× 10^8^**	21	**4.34 × 10^8^**	21	*** 4.34 × 10^8^ ***	16	[5,8,11,12,13,15,16,21,22,23,24]
		1-Propanol	71-23-8	26,439	2.86 × 10^5^	20	**8.41 × 10^6^**	21	*** 1.47 × 10^7^ ***	21	*** 6.06 × 10^6^ ***	16	[3,5,12,16,21,22]
		2-Methyl-1- Propanol	78-83-1	36,856	4.38 × 10^5^	21	**2.05 × 10^6^**	21	*** 5.73 × 10^6^ ***	21	*** 2.36 × 10^6^ ***	15	[5,22]
		1-Butanol	71-363	40,196	6.48 × 10^5^	11	**5.11 × 10^6^**	20	*** 2.84 × 10^7^ ***	21	*** 1.72 × 10^7^ ***	16	[5,9,11,14,15,22,25]
		3-Methyl-1- Butanol ^C^	123-51-3	50,395	6.51 × 10^5^	21	*** 1.65 × 10^7^ ***	21	*** 1.95 × 10^7^ ***	21	*** 1.43 × 10^7^ ***	16	[10,11,25]
	ald.	Octanal	124-13-0	70,320	4.22 × 10^4^	20	**1.41 × 10^5^**	20	*** 1.57 × 10^5^ ***	21	1.01 × 10^5^	14	[23]
		Decanal	112-31-2	76,452	1.40 × 10^5^	19	**5.07 × 10^5^**	21	*** 6.49 × 10^5^ ***	20	4.09 × 10^5^	13	[14,23]
	ket.	2-Heptanone ^C^	110-43-0	63,501	1.09 × 10^5^	21	4.38 × 10^5^	21	**4.71 × 10^5^**	21	*** 5.64 × 10^5^ ***	16	[3,4,7,14,22,27]
	esters	Ethyl Acetate ^C^	141-78-6	33,382	7.54 × 10^5^	21	**1.14 × 10^7^**	21	*** 1.69 × 10^7^ ***	21	*** 2.01 × 10^7^ ***	16	[15,22]
		Ethyl Propionate	105-37-3	46,658	0	0	1.37 × 10^5^	3	**6.06 × 10^5^**	16	*** 2.17 × 10^6^ ***	15	
		Ethyl Butyrate	105-54-4	55,760	0	0	**2.62 × 10^4^**	19	**6.64 × 10^4^**	20	*** 1.60 × 10^5^ ***	16	[7,15]
		n-Butyl Acetate	123-86-4	56,755	3.85 × 10^4^	11	5.67 × 10^4^	20	**2.11 × 10^5^**	20	*** 3.66 × 10^5^ ***	16	[22]
		3-Methylbutyl Acetate (Banana oil)	123-92-2	62,865	0	0	*** 4.15 × 10^4^ ***	21	**5.54 × 10^4^**	21	**8.01 × 10^4^**	14	
		Ethyl n-Octanoate	106-32-1	75,589	0	0	**5.73 × 10^5^**	20	**1.03 × 10^6^**	20	2.33 × 10^5^	12	
		n-Octyl Acetate	112-14-1	75,906	3.94 × 10^4^	1	**3.03 × 10^5^**	19	*** 7.57 × 10^5^ ***	20	**9.76 × 10^5^**	12	
	VSCs	Mercaptoacetone ^C^	24653-75-6	41,956	8.25 × 10^4^	21	**2.94 × 10^5^**	21	**3.36 × 10^5^**	21	1.46 × 10^5^	16	
		Dimethyl Disulfide ^C^ (DMDS)	624-92-0	47,136	2.19 × 10^7^	21	**4.16 × 10^7^**	21	*** 5.74 × 10^7^ ***	21	3.26 × 10^7^	16	[5,6,14,16,23]
	other	Ethyl Vinyl Ether	109-92-2	23,548	6.09 × 10^3^	19	*** 5.19 × 10^4^ ***	21	**3.35 × 10^4^**	21	*** 6.54 × 10^4^ ***	13	
		Acetic Acid	64-19-7	33.000	9.63 × 10^6^	14	**3.25 × 10^7^**	20	**4.19 × 10^7^**	20	1.62 × 10^7^	11	[3,8,14,15,16,22]
		Indole	120-72-9	77,470	1.34 × 10^5^	20	1.17 × 10^5^	21	3.16 × 10^5^	20	*** 3.78 × 10^7^ ***	16	[3,4,5,6,7,8,9,10,11,12,13,14,15,16,17]
TEMPORARY MAXIMUM	HC	1,3-Butadiene	106-99-0	12,987	1.30 × 10^5^	21	*** 5.60 × 10^5^ ***	21	**2.67 × 10^5^**	21	2.46 × 10^5^	15	
		2-Methyl- 2-Butene	513-35-9	21,456	1.40 × 10^5^	21	**1.95 × 10^6^**	21	*** 2.99 × 10^6^ ***	21	2.96 × 10^5^	15	
		Isoprene ^C^	78-79-5	24,927	2.06 × 10^5^	21	*** 6.82 × 10^6^ ***	21	**2.64 × 10^6^**	21	7.85 × 10^5^	16	[3,9,15,21]
		2-Pentene (Z-) ^C^	627-20-3	25,271	6.69 × 10^4^	21	**4.62 × 10^6^**	21	*** 7.97 × 10^6^ ***	21	3.25 × 10^5^	16	
		2-Pentene (E-) ^C^	627-20-3	25,680	6.68 × 10^4^	21	**4.60 × 10^6^**	21	*** 7.88 × 10^6^ ***	21	3.26 × 10^5^	16	
	alc.	2-Propanol ^C^	67-63-0	22,209	1.11 × 10^7^	21	**1.60 × 10^7^**	21	1.51 × 10^7^	21	1.08 × 10^7^	16	
	ald.	Acetaldehyde ^C^	75-07-0	9,644	9.23 × 10^61^	21	**5.65 × 10^7^**	21	**3.07 × 10^7^**	21	2.44 × 10^7^	16	[11,12,16,21,25]
		Propanal ^C^	123-38-6	18,263	2.09 × 10^5^	21	*** 6.44 × 10^5^ ***	21	**8.02 × 10^5^**	21	1.81 × 10^5^	16	[25]
		2-Butenal ^C^	123-73-9	35,863	6.33 × 10^5^	21	*** 5.44 × 10^6^ ***	21	1.14 × 10^6^	21	1.10 × 10^5^	16	
	ket.	Acetone ^C^	67-64-1	18,266	1.08 × 10^8^	21	1.27 × 10^8^	21	1.23 × 10^8^	21	**5.29× 10^7^**	16	[5,11,12,15,17,23,25,26]
	VSCs	Methanethiol	74-93-1	10,900	2.14 × 10^6^	21	**6.28 × 10^6^**	21	**7.26 × 10^6^**	21	1.99 × 10^6^	15	[11,12,16,22]
		Carbon Disulfide ^C^	75-15-0	20,163	7.98 × 10^5^	21	8.95 × 10^5^	21	1.22 × 10^6^	21	**3.34 × 10^5^**	16	[9,21]
		Dimethyl Sulfide ^C^	75-18-3	20,432	2.12 × 10^5^	21	*** 1.23 × 10^7^ ***	21	**4.14 × 10^6^**	21	1.39 × 10^6^	16	[15,21,23,26]
		Ethyl Methyl Sulfide	624-89-5	32,634	0	0	*** 7.00 × 10^5^ ***	20	*** 2.98 × 10^5^ ***	20	1.08 × 10^4^	6	[23]
		Dimethyl sulfone	67-71-0	59,629	1.56 × 10^5^	12	**6.70 × 10^5^**	21	**5.94 × 10^5^**	21	2.47 × 10^5^	8	
	other	Furan	110-00-9	17,916	5.89 × 10^5^	21	*** 2.02 × 10^6^ ***	21	7.52 × 10^5^	21	5.04 × 10^5^	15	
UPTAKE	HC	iso-Butane ^C^	75-28-5	12,000	4.12 × 10^6^	21	*** 4.23 × 10^5^ ***	21	*** 5.66 × 10^5^ ***	21	*** 3.25 × 10^5^ ***	16	
		2-methyl-1- propene	115-11-7	12,629	3.35 × 10^6^	21	**4.97 × 10^5^**	21	*** 4.47 × 10^5^ ***	21	*** 2.52 × 10^5^ ***	14	
		(Z)-2-Butene	590-18-1	12,766	2.07 × 10^6^	21	*** 6.04 × 10^5^ ***	21	*** 6.58 × 10^5^ ***	21	**6.70 × 10^5^**	15	
		n-Butane	106-97-8	13,378	6.42 × 10^4^	21	**1.77 × 10^4^**	21	**2.84 × 10^4^**	21	*** 2.92 × 10^4^ ***	13	
		E-2-Butene	624-64-6	13,659	3.43 × 10^5^	21	**6.80 × 10^4^**	21	*** 6.33 × 10^4^ ***	21	*** 6.00 × 10^4^ ***	14	
		Benzene ^C^	71-43-2	39,320	3.66 × 10^6^	21	2.64 × 10^6^	21	**2.45 × 10^6^**	21	2.87 × 10^6^	16	
		Toluene ^C^	108-88-3	51,942	9.55 × 10^6^	21	8.86 × 10^6^	21	8.47 × 10^6^	21	**5.77 × 10^6^**	16	[5,22,27]
	ald.	Acrolein	107-02-8	17,158	7.76 × 10^5^	21	7.28 × 10^5^	21	**4.00 × 10^5^**	21	**4.59 × 10^5^**	14	
		Methacrolein ^C^	78-85-3	28,261	1.20 × 10^7^	21	**1.71 × 10^6^**	21	*** 3.44 × 10^5^ ***	21	*** 1.97 × 10^5^ ***	16	
		2-Methylpropanal ^C^	78-84-2	28,588	9.59 × 10^6^	21	**1.73 × 10^6^**	21	*** 3.28 × 10^5^ ***	21	*** 7.56 × 10^4^ ***	16	[9,22]
		2-Ethylacrolein	922-63-4	41,552	2.50 × 10^6^	21	**1.47 × 10^5^**	21	*** 5.30 × 10^4^ ***	20	*** 3.88 × 10^4^ ***	14	
		3-MethylButanal ^C^	590-86-3	42,417	1.07 × 10^8^	21	**2.44 × 10^6^**	21	*** 8.14 × 10^5^ ***	21	*** 3.53 × 10^5^ ***	16	[3,4,5,6,9,22]
		2-Methyl- 2-Butenal ^C^	1115-11-3	47,964	5.46 × 10^6^	21	**1.07 × 10^6^**	21	*** 5.81 × 10^5^ ***	21	*** 4.04 × 10^4^ ***	16	
		Furfural ^C^	98-01-1	53,615	8.09 × 10^5^	21	**3.73 × 10^5^**	21	**2.10 × 10^5^**	21	**4.24 × 10^5^**	16	
		Hexanal ^C^	66-25-1	55,982	1.37 × 10^5^	21	**5.82 × 10^4^**	21	**6.20 × 10^4^**	21	**4.87 × 10^4^**	16	[22]
		Benzaldehyde ^C^	100-52-7	65,584	1.83 × 10^7^	21	**2.43 × 10^6^**	21	*** 7.52 × 10^5^ ***	21	*** 8.48 × 10^5^ ***	16	[3,5,7,17,25]
	ket.	Methyl Vinyl Ketone	78-94-4	30,239	2.11 × 10^5^	21	2.63 × 10^5^	21	**1.03 × 10^5^**	21	2.48 × 10^5^	14	
		2,3-Butanedione ^C^	431-03-8	31,663	2.50 × 10^6^	21	4.08 × 10^6^	21	3.52 × 10^6^	21	**9.48 × 10^5^**	16	[3,10,27]
		2-Butanone ^C^	78-93-3	31,539	1.27 × 10^7^	21	1.23 × 10^7^	21	1.13 × 10^7^	21	**5.07 × 10^6^**	16	[5,9,14,22,25]
		2,3-Pentanedione	600-14-6	46,190	1.67 × 10^5^	20	1.80 × 10^5^	21	1.32 × 10^5^	19	**3.22 × 10^4^**	8	[25]
		3-Penten-2-one, (E) ^C^	625-33-2	48,521	3.63 × 10^5^	21	2.94 × 10^5^	21	1.16 × 10^5^	21	**4.10 × 10^4^**	16	
	esters	Methyl Methacrylate ^C^	80-62-6	45,514	1.13 × 10^6^	21	9.95 × 10^5^	21	8.58 × 10^5^	21	*** 3.51 × 10^5^ ***	16	
	other	2-Methylfuran ^C^	534-22-5	31,814	1.04 × 10^6^	21	**5.61 × 10^5^**	21	**4.68 × 10^5^**	21	**2.92 × 10^5^**	16	
		Pyrrole ^C^	109-97-7	45,243	2.88 × 10^5^	21	2.17 × 10^5^	21	1.83 × 10^5^	21	**8.04 × 10^4^**	16	[15]
		2,3-Dimethylfuran ^C^	14920-89-9	44,854	1.75 × 10^5^	21	1.20 × 10^5^	21	**1.06 × 10^5^**	21	**7.83 × 10^4^**	16	[22]
		N,N-Dimethylformamide ^C^	68-12-2	48,577	4.99 × 10^5^	21	**2.01 × 10^5^**	21	**1.74 × 10^5^**	21	**1.33 × 10^5^**	16	
		2,5-DimethylPyrazine ^C^	123-32-0	61,766	8.90 × 10^7^	21	7.58 × 10^7^	21	**7.27 × 10^7^**	21	8.27 × 10^7^	16	[22,25]

Table legend: VOCs—volatile organic compounds; CAS stands for Chemical Abstract Service, providing a unique number identifying a substance, RT—retention time, Occur.—occurrence, HC—hydrocarbons, alc.—alcohols, ald.—aldehydes, ket.—ketones, VSCs—volatile sulfur-containing compounds; ^C^—core volatilome. T0–T6 refer to sampling points at different times of bacterial growth (see Section 3.1. for more details). A bold peak area value indicates a statistically significant difference (Kruskal–Wallis test) between the timepoint of interest and T0. The underlined italic value of peak area in blue font indicates a statistically significant difference in the analogous experiment with reference to susceptible *E. coli* strains.

## Data Availability

The dataset used and analyzed during the current study is available from the corresponding author upon reasonable request.

## Supplementary Materials

The supporting information can be downloaded at https://www.mdpi.com/article/10.3390/ijms26178191/s1.

## Abbreviations

The following abbreviations are used in this manuscript:

VAP	Ventilator-associated pneumonia
VOC	Volatile organic compound
BAL	Bronchoalveolar lavage
GC-MS	Gas chromatography–mass spectrometry
ECDC	European Centre for Disease Control and Prevention
AST	Antimicrobial susceptibility test
ICU	Intensive care unit
CFU	Colony-forming unit
TSB	Tryptic soy broth
DMS	Dimethyl sulfide
DMDS	Dimethyl disulfide
VSC	Volatile sulfuric compound
Susc./S	Susceptible
Res./R	Resistant
IMI	Imipenem
PBS	Phosphate-buffered saline
HC	Hydrocarbon
Alc.	Alcohol
Ald.	Aldehyde
Ket.	Ketone
MCC-IMS	Multi-capillary column-ion mobility spectrometry
GC-IMS	Gas chromatography–ion mobility spectrometry
PTR-MS	Proton transfer reaction–mass spectrometry
SIFT-MS	Selected-ion flow-tube mass spectrometry
FDR	False discovery rate
VIM	Verona-integron encoded beta-lactamase enzyme
