# Supplementary figures and images for: Profiling of Volatile Metabolites of Escherichia coli Using Gas Chromatography–Mass Spectrometry
Source: Int J Mol Sci. 2025 Aug 23;26(17):8191. doi: 10.3390/ijms26178191 (PMC12427675; doi:10.3390/ijms26178191)

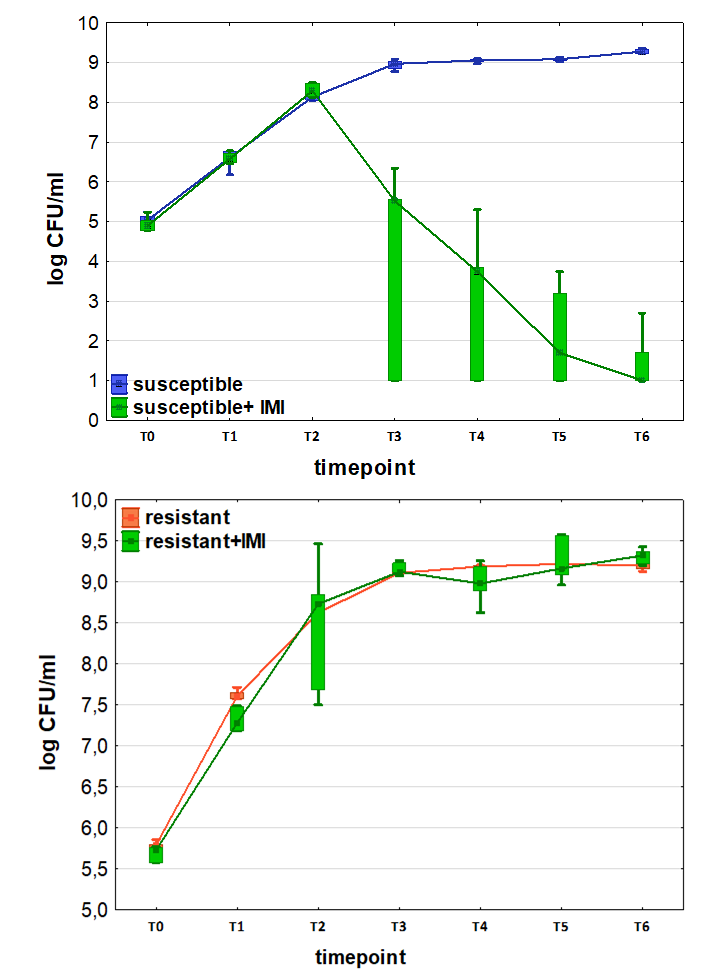

Supplement: Supplementary file 1 [file ijms-26-08191-s001.zip › Figure S1. CFU profiles for sens+IMI_res+IMI.png]

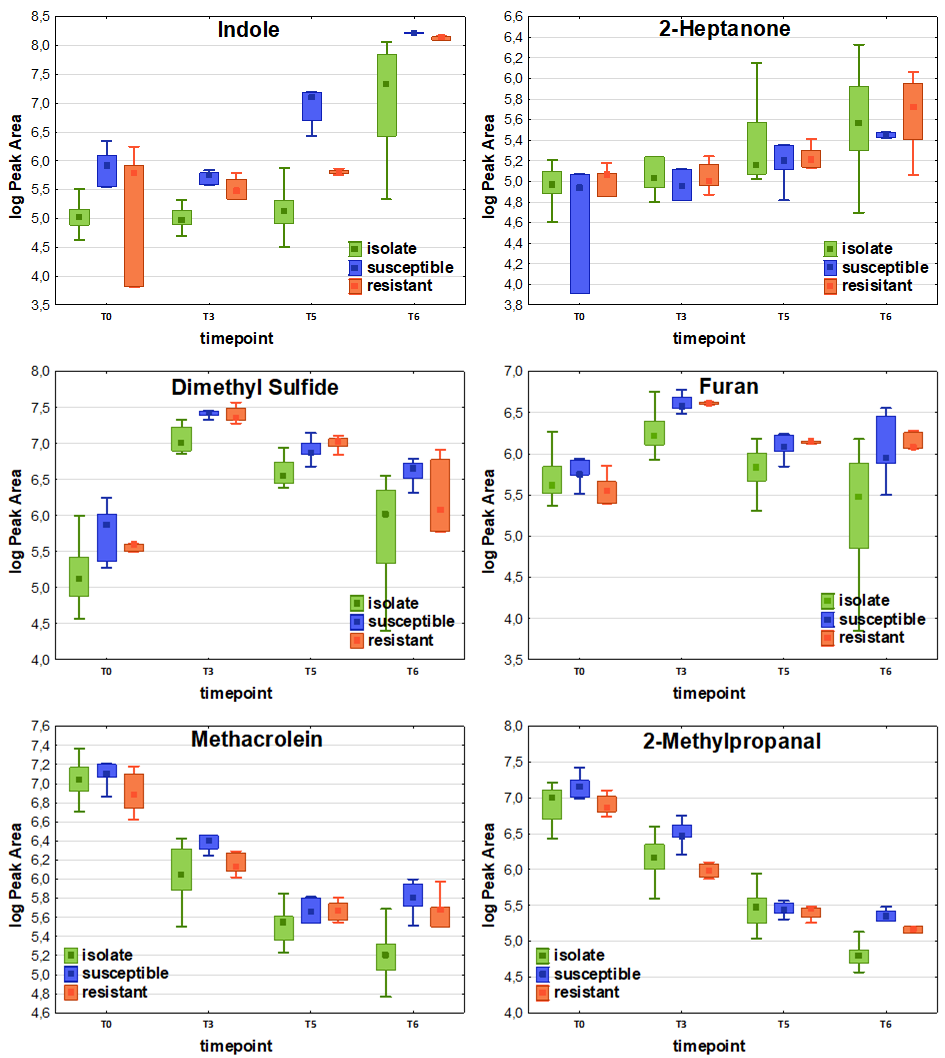

Supplement: Supplementary file 1 [file ijms-26-08191-s001.zip › Figure S2. VOC profiles for isolates.png]
